# The Pentafluorophenyl Cation: A Superelectrophile and Diradical

**DOI:** 10.1002/anie.202512761

**Published:** 2025-08-11

**Authors:** Enrique Mendez‐Vega, Adrián Portela‐González, Ginny Karir, Patrick Hemberger, Wolfram Sander

**Affiliations:** ^1^ Lehrstuhl für Organische Chemie II Ruhr‐Universität Bochum Universitätsstraße 150 44801 Bochum Germany; ^2^ Laboratory for Synchrotron Radiation and Femtochemistry Paul Scherrer Institute (PSI) Villigen CH‐5232 Switzerland

**Keywords:** Carbocations, Fragmentation, Ionization, Pyrolysis, Radicals

## Abstract

We report the first direct observation of the pentafluorophenyl cation in the gas phase via vacuum ultraviolet (VUV) photoionization (PI) of the thermally generated pentafluorophenyl radical. The reactive intermediates and stable reaction products were characterized utilizing photoelectron photoion coincidence (PEPICO) spectroscopy with synchrotron radiation. Electron removal from the pentafluorophenyl radical yields the cation with an adiabatic ionization energy (AIE) of 9.84 ± 0.02 eV. Threshold photoelectron spectra combined with high‐level ab‐initio calculations show that the cation is described as a π^5^σ^1^ diradical with an open‐shell singlet (^1^A_2_) ground state, while the triplet (^3^A_2_) state lies only 1.5 ± 0.4 kcal mol^−1^ higher in energy. The closed‐shell singlet (^1^A) state is highly distorted and lies >4 kcal mol^−1^ above the ground state. This unique aryl carbenium ion exhibits a ∼40 kcal mol^−1^ higher hydride affinity (HA) as compared to the parent phenyl cation, explaining its high reactivity and elusive character. In addition, the radical's reactivity was investigated upon hydrogen abstraction and unimolecular decomposition, forming tetrafluoro *ortho*‐benzyne as well as smaller fluorinated species.

## Graphical abstract

From radical to diradical cation. Photoionization (PI) of the thermally generated pentafluorophenyl radical yields the pentafluorophenyl cation in the gas phase. This cation is found to be a diradical with nearly degenerate open‐shell singlet and triplet ground states, and it is even more unstable than the parent phenyl cation by ∼40 kcal mol^−1^.

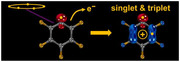

## Introduction

Carbenium ions are crucial intermediates in numerous carbon–carbon bond‐forming reactions. Their reactivity is largely governed by substituents and hybridization, ranging from highly electrophilic species that react with almost any molecule to those that are persistent in solution or the solid state above room temperature. The first report of a persistent organic cation in the early 20^th^ century sparked extensive investigations into the chemical and physical properties of these species.^[^ ^1^ ^]^ Carbenium ions lacking strong electron‐donating substituents are typically highly reactive and short‐lived at room temperature.^[^ ^2^, ^3^ ^]^ Increasing s‐character in the vacant orbital of the carbenium ion correlates with enhanced electrophilicity. Accordingly, the phenyl cation **1a^+^**—a dicoordinated carbocation—is a textbook example of an extremely reactive electrophile.^[^ ^4^, ^5^ ^]^ This ion is prevalent in hydrocarbon plasmas^[^ ^6^ ^]^ and plays a significant role in combustion chemistry.^[^ ^7^ ^]^ It has also been proposed as a precursor in the interstellar synthesis of benzene.^[^ ^8^ ^]^

**Scheme 1:**
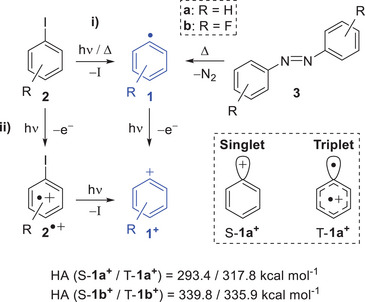
Generation and structure of phenyl cations **1^+^**. HA of phenyl cations **1a^+^** and **1b^+^**, calculated at the G4 level of theory.

Due to its extreme electrophilicity and reactivity, the cation **1a**⁺ eluded direct spectroscopic characterization until the early 2000s, when Sander et al. successfully isolated it in argon matrices at 8 K.^[^ ^9^ ^]^ Cation **1a^+^** was prepared by co‐deposition of iodobenzene **2a** with a microwave‐induced argon plasma and characterized by matrix isolation IR spectroscopy (Scheme 1). Notably, **1a**⁺ was observed to undergo barrierless reactions with N_2_ at 8 K, underscoring its extraordinary electrophilic character.^[^ ^10^ ^]^ The mechanism of formation of **1a^+^** from precursor **2a** involves two possible routes: i) dissociation of the C─I bond (2.83 eV)^[^ ^11^ ^]^ to yield phenyl radical **1a** followed by electron removal with a vertical ionization energy (VIE) of 8.67 eV^[^ ^12^ ^]^ or ii) photoionization (PI) of **2a** to form the corresponding radical cation **2^•+^** (VIE = 8.73 eV)^[^ ^13^ ^]^ and subsequent ion fragmentation. The argon resonance light source with hν = 11.6–11.8 eV provides enough energy to yield cation **1a^+^** by either route i) or ii). A full vibrational assignment and structural characterization of **1a^+^** in the gas‐phase was reported a decade later using Ar‐tagging IRPD spectroscopy, indicating the singlet (^1^A_1_) ground state of **1a^+^**.^[^ ^14^ ^]^ Recently, the IR photofragmentation of **1a^+^** and its perdeutero isotopic cation d_5_‐**1a^+^** was unveiled, pointing to the loss of C_2_H_2_ and C_2_H_4_ as the primary deactivation channels of **1a^+^**.^[^ ^15^ ^]^ Alternatively, radical **1a** can also be produced by thermal decomposition of azobenzene **3a**.^[^ ^16^ ^]^

The adiabatic ionization energy (AIE) of **1a** and the electronic structure of **1a**⁺ have been debated for decades—likely due to broad spectra and limitations in computational accuracy at the time.^[^ ^12^, ^17^, ^18^ ^]^ The singlet cation S‐**1a^+^** (^1^A_1_) possesses a vacant σ orbital at the dicoordinated C1 atom, leading to a wider C6─C1─C2 angle of 145°, shorter C1─C2 bonds, and the partial rehybridization of the C1 atom from sp^2^ to sp.^[^ ^19^ ^]^ In contrast, the triplet state T‐**1a^+^** (^3^B_1_) resembles a diradical/carbene with unpaired electrons in both the σ orbital and the π aromatic ring and a nearly hexagonal geometry (Scheme 1). Large geometrical changes and unfavorable Franck–Condon (FC) factors are predicted for the S‐**1a^+^** ←**1a** vibronic transition, while the opposite is found for the T‐**1a^+^** ←**1a** transition.^[^ ^20^ ^]^ This controversy was solved in 2010 when Stevens et al. determined an accurate AIE of 8.26 eV to the ground state, S‐**1a^+^** by combining PI measurements with thermochemical cycle analysis.^[^ ^21^ ^]^ Very recently, a partially vibrationally resolved photoelectron spectrum of **1a**⁺ was reported by Bentley et al., determining the AIE of the first excited state, T‐**1a^+^**, to be 9.30 eV.^[^ ^22^ ^]^ Combining these spectroscopic data, a singlet–triplet energy gap (Δ*E*_ST_) of −25 kcal mol^−1^ (−1.04 eV) was accurately derived for **1a** **^+^**.^[^ ^22^ ^]^

**Scheme 2:**
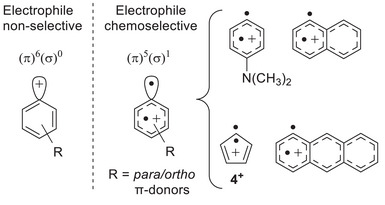
Examples and nature of triplet cations.

The markedly different electronic structure of closed‐shell singlet (π^6^σ^0^) and triplet (π^5^σ^1^) aryl cations confers spin‐dependent reactivity, contributing to their versatility in synthetic chemistry (Scheme 2).^[^ ^23^ ^]^ Singlet cations are indiscriminate electrophiles that react with the solvent and even with N_2_.^[^ ^10^ ^]^ On the contrary, the triplet cations are chemoselective and preferentially attack neutral π nucleophiles (electron‐rich olefins, alkynes, and aromatics), but also charged nucleophiles, such as iodide and cyanide anions.^[^ ^24^, ^25^ ^]^ Singlet aryl cations are typically produced by photocleavage of aryldiazonium salts, while triplet cations are accessible from aryl halides using triplet sensitizers.^[^ ^26^ ^]^ However, the excited state T‐**1a^+^** decays via intersystem crossing (ISC) to S‐**1a^+^** within a few ps time scale in solution, thereby precluding the exploration of its triplet‐state reactivity.^[^ ^23^ ^]^

The triplet state of aryl cations becomes the ground state with strong π‐donor substituents in *para* or *ortho* positions.^[^ ^27^ ^]^ Similarly, β‐substitution of vinyl cations with π‐donors switches the ground state from closed‐shell singlet to triplet.^[^ ^28^ ^]^ Particularly, triplet *p*‐(dialkylamino)aryl cations have been trapped in organic glasses and characterized by EPR and UV spectroscopy (Scheme 2).^[^ ^23^, ^29^ ^]^ Extended conjugation can also lead to a triplet ground state as observed in the naphthyl and anthracenyl cations,^[^ ^30^ ^]^ with the former being characterized by IR spectroscopy in the gas phase.^[^ ^31^ ^]^ The ground state and (anti)aromaticity of the 4π electron cyclopentadienyl cation **4^+^** have long been subjects of debates (see references therein).^[^ ^32^, ^33^ ^]^ The triplet ground state of **4^+^**, trapped in SbF_5_ matrices at 77 K, was demonstrated by EPR spectroscopy.^[^ ^34^ ^]^ The lowest‐energy singlet state, S‐**4^+^**, was reported to lie 4.4 kcal mol^−1^ above T‐**4^+^** in the gas phase using zero‐kinetic energy (ZEKE) photoelectron spectroscopy.^[^ ^35^ ^]^

Fluorine substituents offer a unique synergy of σ‐acceptor and π‐donor character, leading to contrasting effects in singlet (π^6^σ^0^) carbenes.^[^ ^36^ ^]^ For example, the singlet carbene: CF_2_ is quite stable, whereas: C═CF_2_ is an extreme electrophile that readily reacts with N_2_ and Xe even at 10 K.^[^ ^37^, ^38^ ^]^ Perfluorinated carbocations were proposed in the pioneering work of Olah et al.^[^ ^39^ ^]^ as promising reagents in C─F activation and are currently used as synthons in organofluorine chemistry.^[^ ^40^ ^]^ The strong electron‐withdrawing pentafluorophenyl group (─C_6_F_5_) is the backbone of frustrated Lewis acid–base pairs, employed in the metal‐free activation of H_2_ and CO.^[^ ^41^, ^42^ ^]^ Recently, the perfluorinated trityl cation [C(C_6_F_5_)_3_]^+^, along with other halogenated derivatives, was synthesized and structurally characterized at room‐temperature.^[^ ^43^, ^44^ ^]^ These species exhibit high oxidation potentials and hydride ion affinities, as demonstrated by hydride abstraction from alkanes and H_2_.^[^ ^45^ ^]^ The pentafluorophenyl cation **1b^+^** is so electrophilic that it forms a stable crystalline salt with Xe, [XeC_6_F_5_]^+^[B(C_6_F_5_)_4_]^−^. A Xe─C_6_F_5_ bond distance of 2.09 Å was determined by X‐ray crystallography.^[^ ^46^, ^47^ ^]^ However, the cation **1b^+^** has so far eluded direct observation in any media.

**Figure 1:**
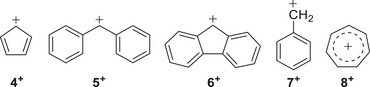
Aryl cations studied in gas and solid phase.

Over the last two decades, we have successfully trapped and spectroscopically characterized highly reactive aryl cations in cryogenic matrices via PI of radicals,^[^ ^9^, ^10^, ^48^ ^]^ protonation of carbenes,^[^ ^49^, ^50^ ^]^ and addition of carbenes to strong Lewis acids.^[^ ^32^ ^]^ Besides the aforementioned **1a^+^**,^[^ ^9^ ^]^ benzhydryl **5^+^**,^[^ ^48^, ^49^ ^]^ fluorenyl **6^+^**,^[^ ^50^ ^]^ as well as a derivative of cation **4^+^** ^[^ ^32^ ^]^ were isolated in cryogenic matrices (Figure 1). However, isolating the pentafluorophenyl cation **1b**⁺ remains a significant challenge due to its extreme electrophilicity. Initial attempts to generate **1b**⁺ in cryogenic matrices were unsuccessful; therefore, we adopted an alternative approach using gas‐phase threshold photoelectron spectroscopy.

Photoelectron photoion coincidence (PEPICO) spectroscopy is an excellent tool to detect and characterize neutral and charged elusive species, allowing the recording of vibrationally‐resolved photoion mass‐selective threshold photoelectron (ms‐TPE) spectra.^[^ ^51^ ^]^ The PEPICO technique coupled to flash‐vacuum pyrolysis (FVP) has been used to investigate fundamental reactive intermediates in the gas‐phase.^[^ ^52^, ^53^ ^]^ In particular, we have studied the resonance‐stabilized benzhydryl **5^+^**,^[^ ^54^ ^]^ fluorenyl **6^+^**,^[^ ^54^ ^]^ benzyl **7^+^**,^[^ ^55^ ^]^ and tropyl **8^+^** cations (Figure 1).^[^ ^56^ ^]^ Experimental vibrational information of their ground and excited states, and the Δ*E*_ST_, was obtained for these aryl cations. This technique is analogous to negative‐ion photoelectron spectroscopy (NIPES), broadly used by Lineberger, Wenthold, and Borden to probe organic anions and upon electron detachment, to experimentally determine the Δ*E*_ST_ of diradicals.^[^ ^57^, ^58^, ^59^ ^]^

In this study, we report for the first time the gas‐phase generation of the highly electrophilic pentafluorophenyl cation **1b**⁺ via PI of the pentafluorophenyl radical **1b**. Radical **1b**, which has never been characterized in the gas phase, is produced by FVP of pentafluoroiodobenzene **2b** and decafluoroazobenzene **3b** (Scheme 1). Furthermore, we elucidate the electronic structure of **1b**⁺ in both its ground and excited states and experimentally determine the singlet–triplet energy gap (Δ*E*_ST_) using a three‐step approach:^[^ ^59^ ^]^ i) theoretical predictions, ii) PI mass spectrometry and ms‐TPE spectroscopy, and iii) calculation‐assisted interpretations of the ms‐TPE spectra.

## Results and Discussion

### Electronic Structure of Pentafluorophenyl Cation 1b^+^

**Figure 2:**
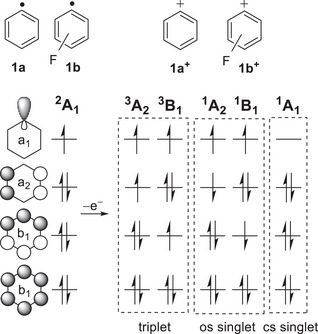
Frontier MOs and lowest‐energy electronic configurations of radicals **1a** and **1b** and their corresponding cations **1a^+^** and **1b^+^**.

**Figure 3:**
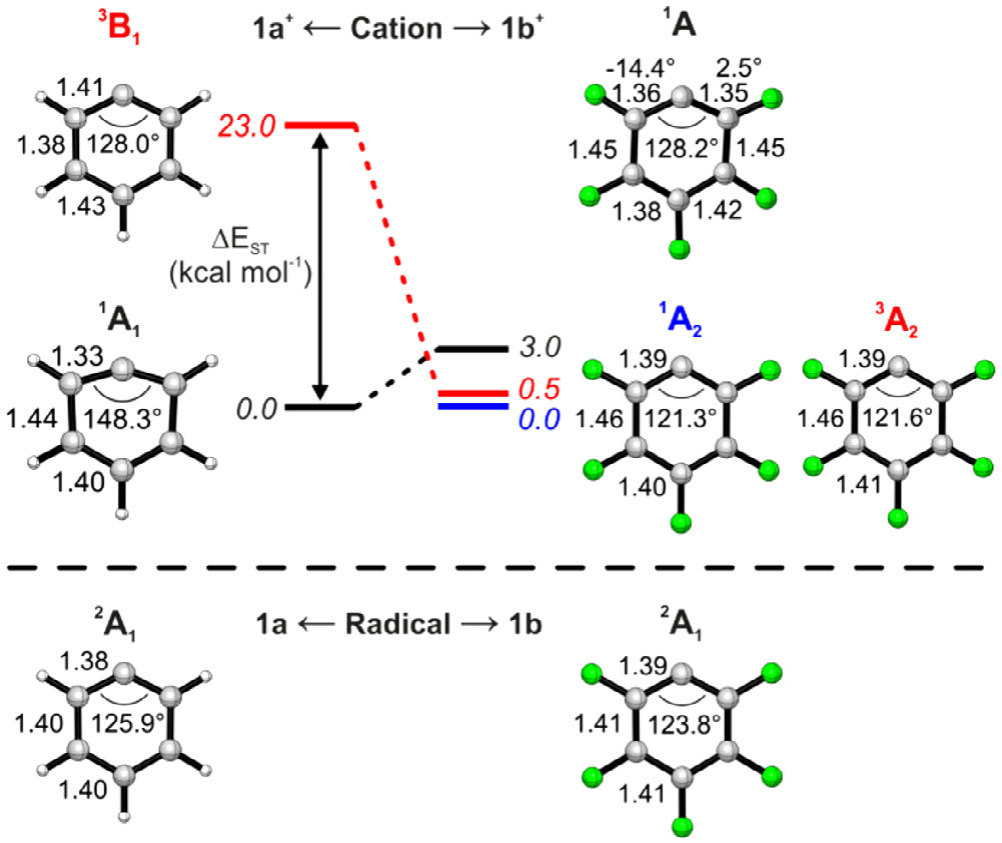
Geometry and relative energies of the lowest‐energy states of radicals **1a** and **1b** and their corresponding cations **1a^+^** and **1b^+^**, optimized at the CASPT2(6/7,7)/aug‐cc‐pVTZ level of theory. The highly distorted **1b^+^** (^1^A) state was optimized with CCSD(T)/aug‐cc‐pVDZ. Selected bond distances (Å), angles (°), and dihedral angles (°) are shown.

To unveil the spectroscopy and reactivity of **1b**, we directly compare it to its non‐fluorinated analogue, **1a**. The aromatic radical **1a** contains three doubly‐occupied π molecular orbitals (MOs) of b_1_, a_2_, and b_1_ symmetry, as well as a singly‐occupied (a_1_) MO, commonly denoted σ (Figure 2). Electron removal of the unpaired electron localized in the σ MO in radical **1a** leads to cation **1a^+^** in its closed‐shell singlet ^1^A_1_ ground state, which exhibits a wider C6─C1─C2 angle of 148°, shorter C1─C2 bonds, and partial rehybridization of the C1 atom from sp^2^ to sp to overcome the electron deficiency. The first excited state, triplet ^3^B_1_, is found to be 25 kcal mol^−1^ higher in energy (Figure 3 and literature),^[^ ^22^ ^]^ while the next higher‐lying states are the isoenergetic ^3^A_2_ and ^1^A_2_ at 30 and 31 kcal mol^−1^, respectively, followed by the ^1^B_1_ state at 39 kcal mol^−1^.^[^ ^19^ ^]^ However, these three higher‐lying states are saddle points and decay to the lowest‐energy ^1^A_1_ and ^3^B_1_ states. Perfluorination of radical **1a** and its cation **1a^+^** results in substantial changes in their electronic structure, geometry, and energetics, as discussed below.

The lowest‐energy electronic states of radical **1b** and its cation **1b^+^** were optimized using single‐configurational DFT, CCSD(T), and composite methods, as well as state‐specific multi‐configurational approaches (Figures 3, S1, and S2; Table S1). In contrast to typical radicals like **1a**, the singly‐occupied σ orbital of **1b** is substantially stabilized, while the doubly‐occupied a_2_ and b_1_ MOs appear at higher energies (Figures S3 and S4). This phenomenon is known as singly occupied molecular orbital–highest occupied molecular orbital (SOMO–HOMO) conversion, and it is a formal violation of the Aufbau principle and orbital ordering.^[^ ^60^ ^]^ Removal of an electron localized in a higher‐energy orbital is associated with a lower ionization energy, according to Koopman's theorem, and typically leads to a relatively more stable state of the corresponding ion. Hence, ejection of an electron from the HOMO a_2_ from radical **1b** results in the formation of the open‐shell singlet ^1^A_2_ and triplet ^3^A_2_ states for **1b^+^**. These states are found to be true energy minima, as demonstrated by frequency calculations, and exhibit a very similar geometry, resembling an allylic structure with long C2─C3 and C5─C6 distances of 1.46 Å and a large separation of the π electron density across the ring (Figure 3). The ^1^A_2_ and ^3^A_2_ states exhibit the lowest energies among all computed states and are nearly degenerate by <0.5 kcal mol^−1^ (Figure 3 and Table S1). Overall, the multi‐configurational methods used indicate an open‐shell singlet ^1^A_2_ ground state, particularly the CASPT2(6,7)/aug‐cc‐pVTZ method that recovers much of both static and dynamic electron correlation (Table S1). While CASPT2 is an expensive method, the results are usually reliable even for complex cases such as open‐shell singlet diradicals and mixed excited states.^[^ ^28^, ^59^, ^61^, ^62^ ^]^ Nevertheless, the geometry and relative energy of the ^3^A_2_ and ^1^A_2_ states are also nicely reproduced by DFT calculations, presumably due to the single‐configuration character of these states (Figures S2 and S5)^[^ ^63^ ^]^ The near‐degeneracy of the ^1^A_2_ and ^3^A_2_ states of **1b^+^** and strong π^5^σ^1^ diradical character are rationalized by the fact that the unpaired electrons are located in the non‐interacting, disjoint a_1_ and a_2_ MOs, with no common atoms.^[^ ^64^ ^]^

Removal of the unpaired electron of radical **1b** yields the cationic ^1^A_1_ (π^6^σ^0^) state, which is a transition state (TS) with two imaginary frequencies corresponding to the in‐plane antisymmetric C─C bond stretch and out‐of‐plane C─C─C bending, leading to a highly distorted C_1_ structure with no symmetry elements (Figures 3 and S1). The distorted ^1^A state lies just 2–4 kcal mol^−1^ above the ^1^A_2_ ground state (Figure 3 and Table S1). Likewise, the higher energy ^3^B_1_ and ^1^B_1_ states are found to be TS with imaginary frequencies corresponding to the in‐plane antisymmetric C─C bond stretch, leading to the lowest‐energy ^3^A_2_ and ^1^A_2_ states (Figure S1 and Table S1).

Overall, it is predicted that perfluorination of the phenyl cation **1a^+^** results in the switching of the closed‐shell (π^6^σ^0^) and open‐shell (π^5^σ^1^) states, leading to near‐degenerated open‐shell singlet (^1^A_2_) and triplet (^3^A_2_) ground states for **1b^+^**. This can be rationalized by the ability of the four F substituents in *ortho* and *meta* positions to jointly stabilize the A_2_ states via π‐electron donation. In contrast, the ^1^A_1_ (π^6^σ^0^) state with a depopulated in‐plane σ orbital cannot benefit from the π‐electron donation, instead, it is destabilized by the strong σ‐acceptor effects of the F substituents. This is somehow mitigated by a highly distorted C_1_ structure, where both the C1 atom and C─F bonds are bent out of the molecular plane. Composite G4 calculations indicate that the energy separation between the triplet and closed shell‐singlet states (Δ*E*_ST_’) of **1a^+^** is reduced by ∼50% by placing an F atom in C4 (*para*) but is completely reversed by double substitution in C2 and C6 (*ortho*). Hence, F substituents in *ortho* positions are found to be the decision‐maker in the spin switching (Figure S4). Our insights into the electronic structure will guide the interpretation of the threshold photoelectron spectra and the exploration of the reactivity in the next sections.

### Gas‐Phase Synthesis of the Pentafluorophenyl Radical 1b

**Scheme 3:**
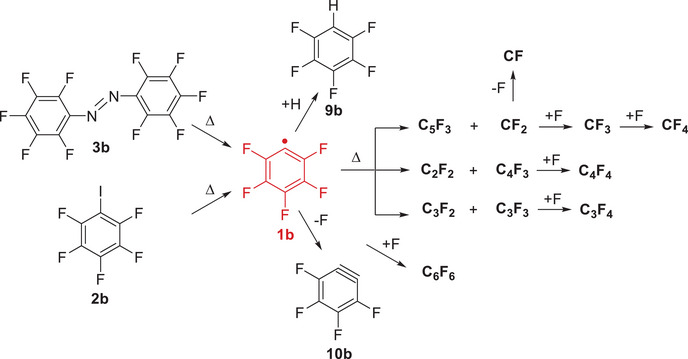
Synthesis and fragmentation of pentafluorophenyl radical **1b** at high temperatures.

**Figure 4:**
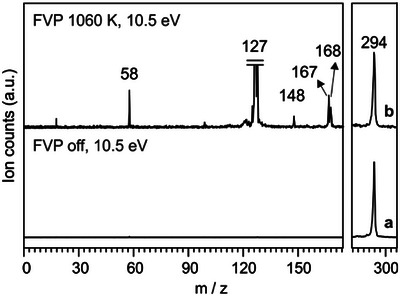
Mass spectra of pentafluoroiodobenzene **2b** at RT (pyrolysis off) and upon FVP at 1060 K with hν = 10.5 eV. Peaks corresponding to detached I atoms (*m/z* 127) are cut off for clarity. The peak at *m/z* 58 arises from acetone contamination in the sample line. Spectrum b (full range) is scaled up by a factor of 50 for clarity.

Radical **1b** was produced by FVP of pentafluoroiodobenzene **2b** and decafluoroazobenzene **3b**, following the thermal route to the parent radical **1a** (Scheme 3).^[^ ^16^ ^]^ The yield of radical **1b** was monitored by recording mass spectra at different photon energies and pyrolysis temperatures (Figures 4 and S6–S8). The collected and mass‐selected photoions were analyzed using velocity map imaging (VMI) to distinguish direct from dissociative ionization (Figure S9).

At RT (pyrolysis off) and a photon energy of 10.5 eV, a single peak appears at *m/z* 294, corresponding to direct ionization of precursor **2b** (Figure 4, trace a). The ion **2b^+^** is photostable from 9 to 13.5 eV, within the typical range of TPE spectroscopy (Figure S6). Pyrolysis of **2b** at 1060 K results in the almost complete depletion of precursor **2b** along with the substantial growth of the peak at *m/z* 167 (Figure 4, trace b). This ion is thus formed via direct ionization of the thermally generated radical **1b** (Figure S9). Additionally, a strong peak at *m/z* 168 appears due to pentafluorobenzene **9b**, which is obtained after efficient H‐abstraction of the very reactive radical **1b** through collisions with the chamber walls (Figure S9). Hence, a relatively clean mass spectrum is obtained upon FVP of **2b** at 1060 K and 10.5 eV (Figure 4, trace b). In addition, a small peak at *m/z* 148 is observed that corresponds to the detachment of an F atom from radical **1b** (Figure S7). This peak is thus assigned to tetrafluoro *ortho*‐benzyne **10b** (see further details in the next section).

To provide an alternative route to radical **1b**, we subjected azo compound **3b** to FVP at 920 K (Figure S8), providing similar yields of the radical at slightly lower temperatures as compared to the iodo precursor **2b**. Isomer‐specific identification of the pyrolysis products **1b** and **10b** and the corresponding ions will be conducted in the following sections by means of ms‐TPE spectroscopy assessed by quantum chemical calculations.

### ms‐TPE Spectrum of the Pentafluorophenyl Radical 1b

**Figure 5:**
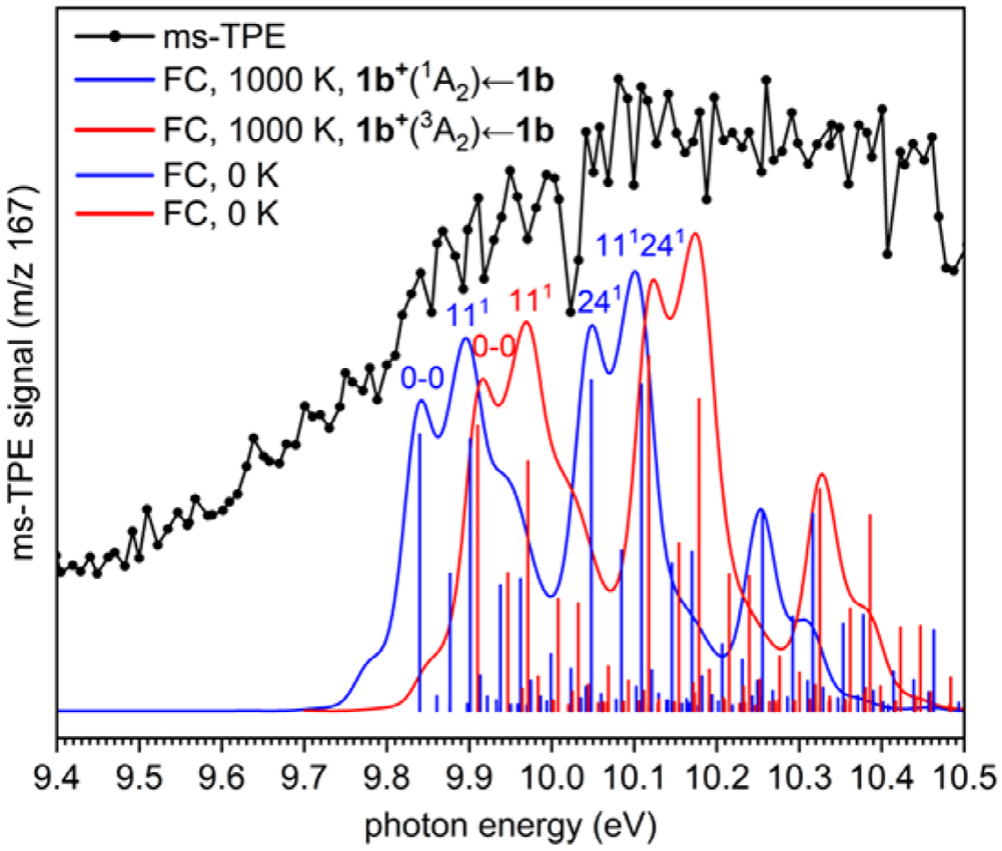
Comparison of the ms‐TPE spectrum of the signal *m/z* 167, recorded upon FVP of precursor **2b** at 1060 K (black trace), with FC simulations at 1000 K of the vibronic transitions of radical **1b** to cation **1b^+^** in its ^1^A_2_ (blue trace) and ^3^A_2_ (red trace) electronic states. FC transitions at 0 K are also shown for the ^1^A_2_ (blue sticks) and ^3^A_2_ (red sticks) states using the CASSCF/aug‐cc‐pVDZ vibrational frequencies. FC simulations are convoluted using 35 meV fwhm Gaussians.

ms‐TPE spectra of the FVP products were recorded by scanning the photon energy and collecting the ms‐TPE signal in coincidence with a selected mass in the ion image. As the signal is mass‐selected, contributions from remaining precursor or thermal byproducts are excluded, in contrast to conventional photoelectron spectroscopy. The ms‐TPE spectrum of *m/z* 167, obtained after FVP of precursor **2b** at 1060 K is depicted in Figure 5.

The ms‐TPE spectrum shows a broad band system with two characteristic regions at 9.8–10.0 and 10.0–10.2, followed by a long tail above 10.2 eV (Figure 5). The first asymmetric band starts rising slowly from 9.7 eV due to a congested set of hot bands (Figure S10), which is typical for rovibrationally hot phenyl ring species, as recently shown.^[^ ^65^ ^]^ A series of structured peaks starting between 9.84 and 10.00 eV consistently appear in the spectra obtained upon FVP of **2b** at different temperatures but also upon FVP of precursor **3b** (Figure S10), which lends further proof to our assignment of radical **1b** formed via FVP of both precursors. As discussed in the previous section, only three of the lowest‐energy states computed are true minima: the planar and near‐degenerate ^1^A_2_ and ^3^A_2_ states and the highly distorted ^1^A state lying at higher energies (Figure 3). Hence, the peaks at 9.84 and 9.91 ± 0.02 eV are assigned to the AIEs of the cation **1b^+^** in its lowest‐energy ^1^A_2_ and ^3^A_2_ states, respectively (Table 1). Systematic recovering of the electron correlation, as in the computational methods presented in Table 1, leads to AIEs to the ^1^A_2_ and ^3^A_2_ states of about 9.8–9.9 eV, in excellent agreement with our experiment. This allows determining the singlet–triplet gap of **1b^+^** to −0.07 ± 0.02 eV (−1.5 ± 0.4 kcal mol^−1^), which is reasonably well reproduced by CASPT2/aug‐cc‐pVTZ computations (Tables 1 and S1).

**Table 1:** Experimental and calculated AIEs of **1b** in eV.

Methods	AIE^a)^ (^1^A_2_)	AIE^b)^ (^3^A_2_)	AIE^c)^ (^1^A)	Δ*E*_ST_ ^d)^
ms‐TPES (exp.)	9.84	9.91	>10	−0.07
B3LYP/6–311+G*^e)^	9.70	9.72	10.10	−0.02
NEVPT2/aug‐cc‐pVTZ	9.73	9.72	–	+0.01
CIPT2/aug‐cc‐pVTZ	9.75	9.75	–	0
CASPT2/aug‐cc‐pVTZ	9.80	9.82	–	−0.02
CCSD(T)/aug‐cc‐pVDZ	–	9.80	9.94	–
CCSD(T)/aug‐cc‐pVTZ	–	9.83	9.90	–
G4^e)^	–	9.84	10.01	–
W1BD^e)^	–	9.91	10.03	–

^a)^ Transition **1b^+^** (^1^A_2_) ← **1b**.

^b)^ Transition **1b^+^** (^3^A_2_) ← **1b**.

^c)^ Transition **1b^+^** (^1^A) ← **1b**.

^d)^ Singlet–triplet energy gap (Δ*E*_ST_) of **1b** ^**+**^.

^e)^ DFT and composite calculations include zero‐point energy (ZPE) corrections.

To further proof our assignment, we conducted FC simulations of the vibronic transitions from radical **1b** to cation **1b^+^** in its ^1^A_2_ and ^3^A_2_ states at 1000 K, which reasonably reproduce the spectral pattern: i) the band splitting for both the ^1^A_2_ and ^3^A_2_ states between 9.8 and 10.0 eV; and ii) the cut‐off in the spectrum at 10.0 eV leading to the second characteristic region at 10.0–10.2 eV. Such a complex spectral pattern (Figure 5) arises from the overlapping of vibrational progressions involving the ν_11_ (ring breathing) and the ν_24_ (C═C stretching) vibrational modes for both geometrically similar ^1^A_2_ and ^3^A_2_ states (Figure 3). The discrepancy between the FC simulation and the experimental spectrum may be explained by an increase of the threshold PI cross sections of the hot band transitions.^[^ ^65^ ^]^ Nevertheless, FC simulations of the **1b^+^** (^3^A_2_) ← **1b** vibronic transition show no difference regardless of the chosen method, whether CASSCF, NEVPT2, DFT, or CCSD(T) (Figure S11). Fitting of the experimental spectra with FC simulations (Figure 5) allows determining vibrational frequencies of ∼0.05 ± 0.02 eV (400 ± 160 cm^−1^) and ∼0.20 ± 0.02 eV (1530 ± 160 cm^−1^) for the ν_11_ and ν_24_ modes of cation **1b^+^** in the geometrically similar ^1^A_2_ and ^3^A_2_ states. In agreement, vibrational frequencies of 464 and 1528 cm^−1^ are estimated for the ν_11_ and ν_24_ modes of the **1b^+^** (^3^A_2_) state with NEVPT2/aug‐cc‐pVDZ. On the other hand, the **1b^+^** (^1^A) ← **1b** vibronic transition exhibits very poor FC factors due to the extreme geometrical change; hence, it is predicted to contribute to the broad tail above 10 eV, according to G4 and W1BD composite calculations. A similar scenario was recently reported for the non‐classical ethyl^[^ ^66^ ^]^ or vinyl cations.^[^ ^67^ ^]^ In summary, we explored the electronic structure of the pentafluorophenyl radical **1b** and cation **1b^+^** and the differences from its parent, phenyl **1a,** using a combined experimental and theoretical approach.

### Reactivity of the Pentafluorophenyl Radical 1b and its Cation 1b^+^

In this section we discuss the reaction of radical **1b** with H atoms to form pentafluorobenzene **9b** and its thermal decomposition to yield tetrafluoro‐*o*‐benzyne **10b** via defluorination. The ms‐TPE spectrum of **9b** resembles that of radical **1b** with two overlapping vibrational progressions, which is indicative of perfluorinated aromatic compounds. The AIE of **9b** was determined to be 9.64 ± 0.02 eV using different experimental conditions and precursors and is in good agreement with results from G4 (9.60 eV) and CCSD(T)/aug‐cc‐pVTZ (9.58 eV) calculations (Figure S12).

**Scheme 4:**
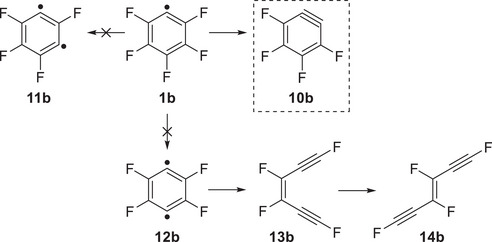
Synthesis and interconversion between C_6_F_4_ isomers.

The ms‐TPE spectrum of the signal at *m/z* 148, recorded upon FVP of **2b** at 1200 K, shows a broad spectrum with an onset starting at 9.9 eV (Figure S13). The experimental AIE of 9.95 eV is in good agreement with the computed AIE of tetrafluoro‐*o*‐benzyne **10b** (9.9 to 10.0 eV depending on the computational method; see Table S3). In contrast to *o*‐benzyne **10a** ^[^ ^61^ ^]^ the ground state of the perfluorinated benzyne **10b** is found to be planar (^2^A_2_). However, excited states of **10b** might contribute to the TPE spectrum at higher energies. Isomeric perfluorinated *meta*‐ and *para*‐benzynes **11b** and **12b**,^[^ ^68^ ^]^ as well as the acyclic 1,3,4,6‐tetrafluorohex‐3‐ene‐1,5‐diynes **13b** and **14b** ^[^ ^69^ ^]^ (Scheme 4), exhibit lower AIEs of 9.1–9.7 eV and do not seem to be present in the gas phase (Figure S13 and Table S3). Benzyne isomers **11b** and **12b** are calculated to lie 9 and 33 kcal mol^−1^ higher in energy than the *ortho* isomer, while the acyclic **13b** and **14b** are even more unstable at 36 kcal mol^−1^ (Table S3). In contrast to the parent system (**12a** → **13a**), the ring‐opening of the *para*‐benzyne isomer (**12b** → **13b**) is found to be endothermic with a high energy barrier due to the drastic destabilization of alkynes by fluorine substituents at the terminal acetylenic positions.^[^ ^69^ ^]^

The thermal window for producing radical **1b** is restricted to 1000–1200 K (Figure S7). Further increase of the FVP temperature yields a variety of smaller C_n_F_m_ fragments (listed in the Supporting Information), which are visible at higher photon energies of 13.5 eV and assigned by comparison to data from electron ionization of hexafluorobenzene (C_6_F_6_).^[^ ^70^ ^]^ High‐energy isomers of C_6_F_6_ like 1,2,3‐cyclohexatriene are ruled out (Scheme 3 and Figure S8). C_6_F_6_, C_4_F_4_, C_5_F_3_, C_3_F_3_, CF_2_, and F atoms are thermally produced upon FVP of **2b** at 1060 K, as observed at hν = 13.5 eV. Accordingly, C_5_F_3_ (*m/z* 117) and CF_2_ (*m/z* 50) are formed from radical **1b** (*m/z* 167), while the addition or loss of an F atom (*m/z* 19) leads to CF_3_ (*m/z* 69) or CF (*m/z* 31), respectively (Scheme 3 and Figure S6). This explains why earlier attempts to thermally generate radical **1b** from precursor **2b** were not successful, due to the complete breakdown of the aromatic ring.^[^ ^71^ ^]^ Extensive thermal fragmentation was also found in the unsuccessful thermal preparation of the fluorinated benzyne **10b**.^[^ ^68^ ^]^

**Figure 6:**
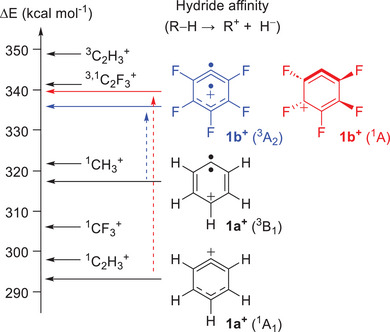
HA of the closed‐shell singlet (csS) and triplet (T) states of (perfluorinated) methyl (CH_3_^+^), vinyl (C_2_H_3_^+^), and phenyl cations **1a^+^** and **1b^+^**, calculated at the G4 level of theory (in kcal mol^−1^). C_2_F_3_^+^ is found to have nearly degenerated csS and T states.

Experimental spectra, supported by high‐level calculations, reveal a contrasting electronic structure for the perfluorinated cation **1b^+^** compared to the parent cation **1a^+^** (Figure 3). These observations raise important questions: i) is the singlet–triplet state switching from the parent **1a^+^** to **1b^+^** due to stabilization of the diradical ^1^A_2_ and ^3^A_2_ states or a destabilization of the closed‐shell singlet (^1^A) state? ii) how reactive is the perfluorinated cation **1b^+^**? To address these questions, we compute the hydride affinity (HA), an established measure of carbocation thermodynamic stability.^[^ ^72^ ^]^ Since the open‐shell ^1^A_2_ and ^3^A_2_ states are nearly degenerate, and for the sake of simplicity, we only examine the lowest‐energy triplet versus the closed‐shell singlet ground state in cations **1a^+^** and **1b^+^** at the G4 level of theory (Figure 6 and Table S2).

Computed HA at the G4 level indicates that, for the parent cation **1a⁺**, the triplet (^3^B₁) state lies 25 kcal mol^−1^ above the closed‐shell singlet (^1^A_1_) ground state (Figure 6 and Table S2). Perfluorination of **1a⁺** leads to a significant destabilization of the singlet state by 47 kcal mol^−1^, while the triplet state is comparatively less affected (red and blue dashed arrows in Figure 6), resulting in a switching of the spin ground‐state multiplicity in **1b⁺**. A similar trend is predicted for the vinyl cation (C_2_H_3_^+^): perfluorination selectively destabilizes the closed‐shell singlet state of C_2_H_3_^+^, leading to nearly degenerate singlet and triplet ground states in C_2_F_3_^+^.^[^ ^28^ ^]^

An additional indication of the extreme reactivity of cation **1b⁺** is its strong binding to N_2_, yielding the pentafluorophenyl diazonium ion [**1b–**N_2_]⁺. This species exhibits a calculated dissociation energy of 48 kcal mol^−1^ at the G4 level, much higher than that of the parent [**1a–**N_2_]⁺ (29 kcal mol^−1^), indicating significantly greater stability.^[^ ^4^ ^]^ Taming the energetic phenyl diazonium ions has long been a goal in the pursuit of controlled N_2_ extrusion, thus enabling safer and more efficient Sandmeyer reactions.^[^ ^73^ ^]^

## Conclusion

The dicoordinated phenyl cation **1a⁺** plays a central role as a reactive intermediate in organic synthesis and under high temperatures and ionizing conditions. However, its exceptional electrophilicity and reactivity have challenged the determination of its physical and chemical properties for nearly a century. Perfluorination of aryl cations has recently proven to be an effective tool for generating superelectrophiles, as termed by Olah,^[^ ^74^ ^]^ with increased HA,^[^ ^43^ ^]^ capable of activating molecular hydrogen and short‐chain alkanes.^[^ ^45^ ^]^ The pentafluorophenyl cation **1b⁺**, with an HA ∼40 kcal mol^−1^ higher than that of the parent cation **1a⁺**, is so reactive that it even activates xenon to form a crystalline isolable cation [XeC_6_F_5_]^+^.^[^ ^46^, ^47^ ^]^ In contrast to **1a^+^**, the perfluorinated cation **1b^+^** has, so far, eluded detection or isolation in any medium.

In this work, we report the first direct observation and structural characterization of the elusive cation **1b^+^** in the gas phase via vacuum ultraviolet (VUV) PI of the thermally generated radical **1b**. Ms‐TPE spectra combined with high‐level CASPT2/aug‐cc‐pVTZ calculations reveal that cation **1b^+^** exhibits a unique electronic structure with near‐degenerate open‐shell singlet and triplet ground states separated by ∼1.5 kcal mol^−1^ (0.07 eV), resembling a π^5^σ^1^ diradical. Our approach is antagonistic to the strategy proposed by Chen et al. in stabilizing the σ^0^π^6^ singlet state in cyclohexa‐2,5‐dienylidenes using σ‐donor and π‐acceptor substituents.^[^ ^62^ ^]^ The unusual combination of carbocation and diradical nature makes **1b^+^** a promising chemoselective reactive intermediate in organic synthesis.^[^ ^28^, ^75^ ^]^

Finally, radical **1b** serves as an extremely reactive but versatile probe, undergoing various high‐temperature reactions, including: i) hydrogen abstraction to form pentafluorobenzene **9a**; ii) fluorine detachment to yield tetrafluoro‐*o*‐benzyne **10b**; and iii) complete fragmentation of the aromatic ring into small C_n_F_m_ species such as C_5_F_3_, C_3_F_3_, CF_2_, and F atoms. Once again, the synergy between ms‐TPE spectroscopy and advanced computational methods has enabled the isomer‐specific identification of elusive species, providing valuable insights into their electronic, vibrational, and geometric structures. These results offer important benchmarks for computational chemistry and contribute to the development of the Active Thermochemical Tables (ATcT),^[^ ^76^ ^]^ as exemplified by the recent spectroscopic study of the phenyl cation **1a⁺** ^[^ ^22^ ^]^ and the comprehensive work presented here on the pentafluorophenyl radical **1b** and its cation **1b^+^**.

## Supporting Information

The authors have cited additional references within the Supporting Information.^[^ ^77^, ^78^, ^79^, ^80^, ^81^, ^82^, ^83^, ^84^, ^85^, ^86^, ^87^, ^88^ ^]^

## Conflict of Interests

The authors declare no conflict of interest.

## Supporting information



Supporting Information

## Data Availability

The data that support the findings of this study are available from the corresponding author upon reasonable request.
