# Supplementary material for: The Pentafluorophenyl Cation: A Superelectrophile and Diradical
Source: Angew Chem Int Ed Engl. 2025 Aug 11;64(39):e202512761. doi: 10.1002/anie.202512761 (PMC12455445; doi:10.1002/anie.202512761)
Supplement: Supplementary file 1 — Supporting Information [file ANIE-64-e202512761-s001.docx]

**The Pentafluorophenyl Cation: A Superelectrophile and Diradical**

Enrique Mendez-Vega^[a]^, Adrián Portela-González^[a]^, Ginny Karir^[a]^, Patrick Hemberger^[b]^ *, and Wolfram Sander^[a]^ *

^[a]^ Lehrstuhl für Organische Chemie II, Ruhr-Universität Bochum, 44780 Bochum, Germany

^[b]^ Laboratory for Femtochemistry and Synchrotron Radiation, Paul Scherrer Institut (PSI), CH-5232 Villigen, Switzerland

Table of Contents

[Experimental Details S2](#_Toc200406359)

[Quantum Chemical Calculations S3](#_Toc200406360)

[Mass Spectra of the FVP Products S7](#_Toc200406361)

[Velocity Map Imaging S11](#_Toc200406362)

[ms-TPE Spectra of the FVP Products S12](#_Toc200406363)

[Cartesian Coordinates S16](#_Toc200406364)

[References S18](#_Toc200406365)

# Experimental Details

**Experiments.** Experiments were performed utilizing the double imaging photoelectron photoion coincidence (CRF-PEPICO) spectrometer at the VUV beamline of the Swiss Light Source located at Paul Scherrer Institute.^[1-2]^ Pentafluoro azobenzene **3b** was synthesized according to the literature^[3]^ and pentafluoroiodobenzene **2b** is commercially available. Precursors **2b** and **3b** were sublimed along with a He flow of 30–40 sccm, and the gas mixture subsequently expanded through a 100 μm nozzle into a pyrolysis reactor. The reactor consist of a 40 mm long SiC tube with an inner diameter of 1 mm and an electrically heated length of 15 mm.^[4]^ The pressure and the residence time inside the reactor is estimated to be 10–20 mbar and ∼25–50 μs.^[5]^ After skimming (2 mm), the molecular beam reaches the experimental chamber and is ionized by tunable VUV synchrotron radiation. During experiments, the pressure in the source and experimental chamber is about 3 × 10^–5^ and 3 × 10^–6^ mbar, respectively.

After ionization, electrons and ions are vertically extracted in opposite directions via an electric field (218 V/cm). The photoions are analyzed using time-of-flight mass spectrometry and velocity map imaging (VMI), which enables to distinguish the molecular beam (MB) emanating from the hot FVP reactor from the rethermalized background (BG) signals, as well as direct ionization (DI) from dissociative photoionization (DPI).^[6]^ Threshold electrons with a kinetic energy of <10 meV and photoions are selected in coincidence to record mass-selected threshold photoelectron (ms-TPE) spectra.^[7]^ Spectra were corrected for false coincidences and the hot electron contribution was subtracted using the approach by Sztaray and Baer.^[8]^ The ms-TPE spectra were recorded using a photon energy step scan of 10 meV, resulting in uncertainties of ±0.01 eV for the reported AIEs.

**Calculations.** Geometries and vibrational frequencies of the neutral and cationic species in their ground and excited states were initially computed at the B3LYP/6-311+G* level of theory with the programs Gaussian 16^[9]^ and Turbomole 7.5.^[10]^ Adiabatic ionization energies (AIE) and singlet-triplet energy gaps (ΔE_ST_) were refined with the CBS-QB3, G4, and W1BD composite methods.^[11]^ ΔE_ST_ refers to the gas-phase energy difference between the lowest-energy singlet and triplet states, with or without zero-point energy (ZPE) corrections. A negative value for ΔE_ST_ indicates a singlet ground state.

For a rigorous treatment of multi-configurational systems and proper recovering of both static and dynamic electron correlation,^[12-15]^ the lowest-energy states of radical **1b** and cation **1b^+^** were computed with CASSCF, CIPT2, NEVPT2, CASPT2, as well as CCSD(T) (for single-configurational states) using correlation-consistent (aug)-cc-pV(D/T)Z basis sets. In the state-specific multiconfigurational calculations, the active space (CAS) consisted of the σ, low-lying filled and empty π orbitals as well as the corresponding electrons, denoted CAS(7,7) for **1b** and CAS(6,7) for **1b^+^**. Coupled cluster and multi-configurational calculations were conducted with Molpro 2012.^[16]^ Franck–Condon simulations at 0, 300, and 1000 K using the vibrational frequencies computed with different methods, were performed with the program ezSpectrum.^[17]^ The stick spectra were subsequently convoluted with a Gaussian function (fwhm = 35 meV).

# Quantum Chemical Calculations

Table S1. Calculated Adiabatic Ionization Energies (AIE) from 1b to 1b^+^ in its Lowest-Energy Electronic States (in eV)

| **Method** | **^3^A_2_ (C_2v_)** | **^1^A_2_ (C_2v_)** | **^3^B_1_ (C_2v_)** | **^1^B_1_ (C_2v_)** | **^1^A_1_ (C_2v_)** | **^1^A (C_1_)** |
| --- | --- | --- | --- | --- | --- | --- |
| B3LYP/6-311+G* | 9.72 | –0.02 | +0.19 | +0.42 | +1.21 | +0.39 |
| B3LYP/6-311+G* + ZPE | 9.70 | –0.02 | +0.15 | +0.34 | +1.23 | +0.41 |
| CASSCF/aug-cc-pVDZ | 9.43 | –0.08 | 0 | +0.72 | +1.30 | – |
| CASSCF/aug-cc-pVDZ + ZPE | 9.40 | –0.05 | – | – | – | – |
| CASSCF/aug-cc-pVTZ | 9.30 | –0.08 | +0.02 | +0.74 | +1.29 | – |
| NEVPT2/aug-cc-pVTZ | 9.72 | +0.01 | +0.19 | +0.71 | +1.33 | – |
| CIPT2/aug-cc-pVTZ | 9.74 | 0 | +0.18 | +0.72 | +1.47 | – |
| CASPT2/aug-cc-pVDZ | 9.78 | –0.02 | +0.06 | +0.63 | +1.50 | – |
| CASPT2/aug-cc-pVTZ | 9.81 | –0.02 | +0.06 | +0.59 | +1.44 | – |
| CCSD(T)/cc-pVDZ | 9.44 | – | +0.20 | – | +1.15 | +0.11 |
| CCSD(T)/cc-pVDZ + ZPE | 9.46 | – | +0.14 | – | +1.10 | +0.09 |
| CCSD(T)/cc-pVTZ | 9.67 | – | +0.21 | – | +1.07 | +0.08 |
| CCSD(T)/aug-cc-pVDZ | 9.80 | – | +0.17 | – | +1.13 | +0.14 |
| CCSD(T)/aug-cc-pVTZ//ADZ | 9.83 | – | +0.21 | – | +1.06 | +0.07 |
| CBS-QB3 | 9.86 | – | +0.12 | – | – | +0.09 |
| G4 | 9.84 | – | +0.14 | – | – | +0.17 |
| W1BD | 9.91 | – | +0.13 | – | – | +0.12 |
| Stationary Point | Min. | Min. | TS | TS | TS | Min. |

Calculated AIE from **1b** to **1b^+^** (^3^A_2_), this state is used as reference since it can be calculated with all methods. Calculated energies of the lowest-energy electronic states of **1b^+^** with respect to the ^3^A_2_ state. The open-shell singlet states (^1^A_2_ and ^1^B_1_) are calculated with multiconfigurational calculations and DFT (using a broken-symmetry wave function). Composite calculations include zero-point energy (ZPE) corrections.

Table S2. Hydride Affinity (R–H → R^+^ + H^–^) for Selected Cations in their Lowest-Energy Electronic States Calculated with G4 (in kcal mol^-1^)

| **Cation** | **closed-shell Singlet** | **Triplet** | **ΔE_ST_’** |
| --- | --- | --- | --- |
| **1a^+^** | 293.4 | 317.8 | –24.4 |
| **1b^+^** | 339.8 | 335.9 | +3.9 |
| **CH_3_^+^** | 321.7 | – | – |
| **CF_3_^+^** | 306.1 | – | – |
| **C_2_H_3_^+^** | 298.8 | 348.5 | –49.7 |
| **C_2_F_3_^+^** | 341.3 | 342.0 | +0.7 |


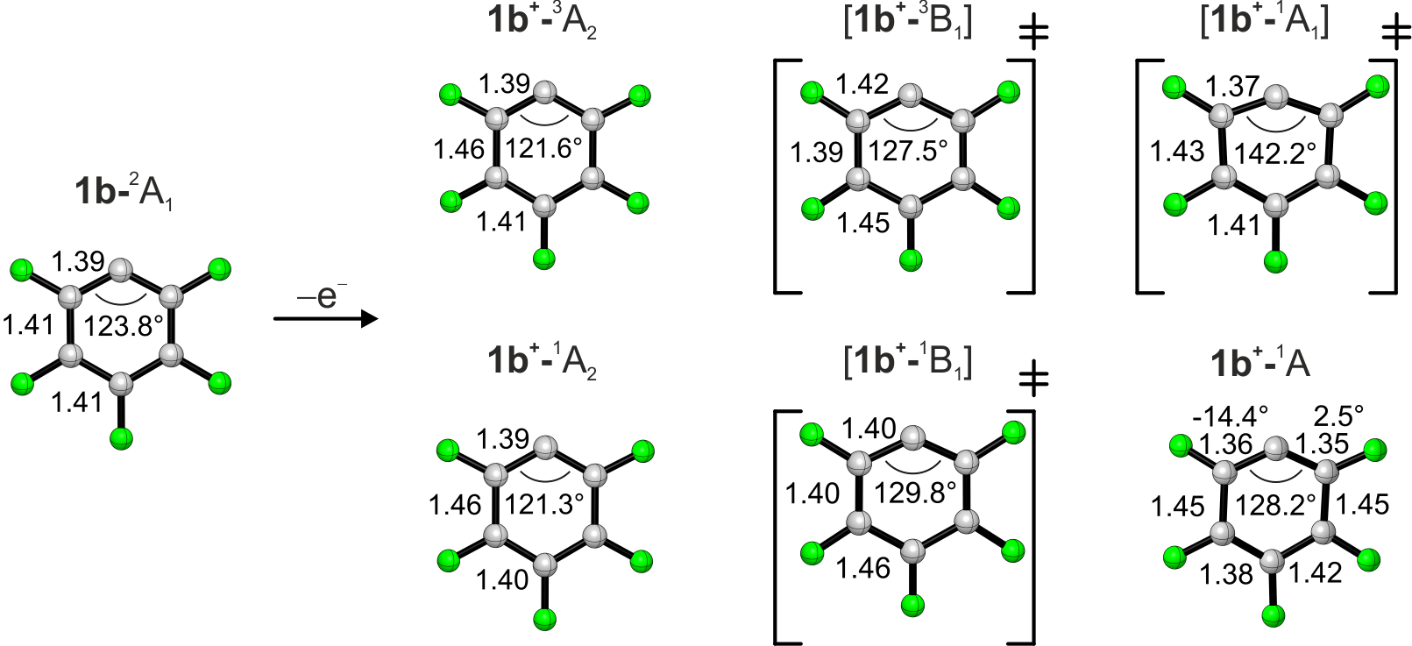


**Figure S1.** Selected bond distances (Å), angles (°), and dihedral angles (°) of the lowest-energy electronic states of **1b^+^**, optimized at the CASPT2(6,7)/aug-cc-pVTZ level of theory. The highly distorted **1b^+^** (^1^A) state was optimized with CCSD(T)/aug-cc-pVDZ. Transition states are shown in square brackets. Imaginary frequencies corresponding to the in-plane antisymmetric C–C bond stretch are found for the ^3^B_1_, ^1^B_1_, and ^1^A_1_ states while that assigned to the out-of-plane C­–C–C bending also appears in the ^1^A_1_ state.


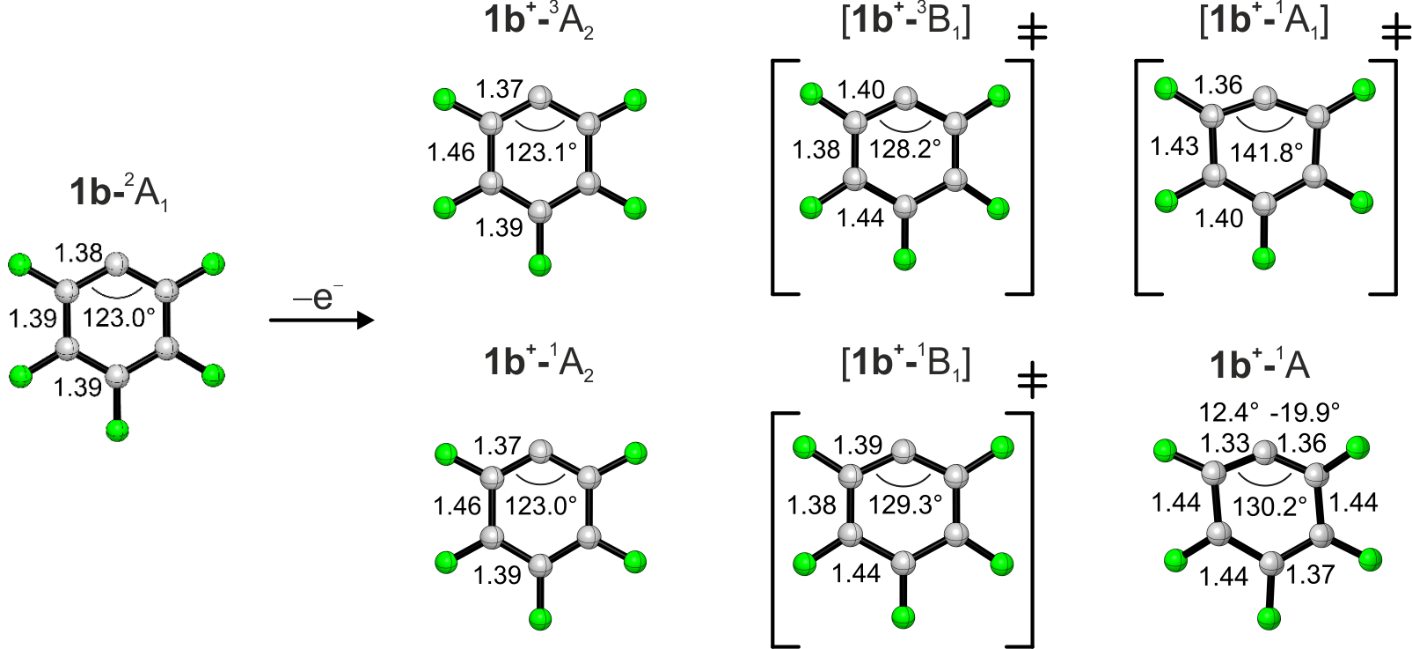


**Figure S2.** Selected bond distances (Å), angles (°), and dihedral angles (°) of the lowest-energy electronic states of **1b^+^**, optimized at the B3LYP/6-311+G* level of theory. Transition states are shown in square brackets. Imaginary frequencies corresponding to the in-plane antisymmetric C–C bond stretch are found for the ^3^B_1_, ^1^B_1_, and ^1^A_1_ states while that assigned to the out-of-plane C­–C–C bending also appears in the ^1^A_1_ state.

**Figure S3.** Effect of fluorination on the highest-energy singly- (a_1_) and doubly-occupied (a_2_ and b_1_) molecular orbitals (MOs) of phenyl radical **1a**. Absolute energies of the MOs given in Hartrees, and calculated at the B3LYP/6-311+G* level of theory.

**Figure S4**. Effect of F substitution on the geometry of the closed shell-singlet state of **1a^+^**, and its energy gap with the triplet state (ΔE_ST_’), calculated at the G4 level of theory (in kcal mol^-1^). Negative ΔE_ST_’ indicates a closed-shell singlet ground state.

**Figure S5.** Orbital energy diagram for leading configurations of pentafluorophenyl radical **1b** and its cation **1b^+^** in the five lowest energy electronic states (C_2v_) optimized at the CASPT2(6/7,7)/aug-cc-pVTZ level of theory. Occupation numbers of natural orbitals are given in italic and the CI coefficient (Ci) of the respective configuration in the reference wave function is given in bold. The open-shell singlet ^1^a_2_ and ^1^B_1_ states are described by two configurations with the same weight but alternating spins.

# Mass Spectra of the FVP Products


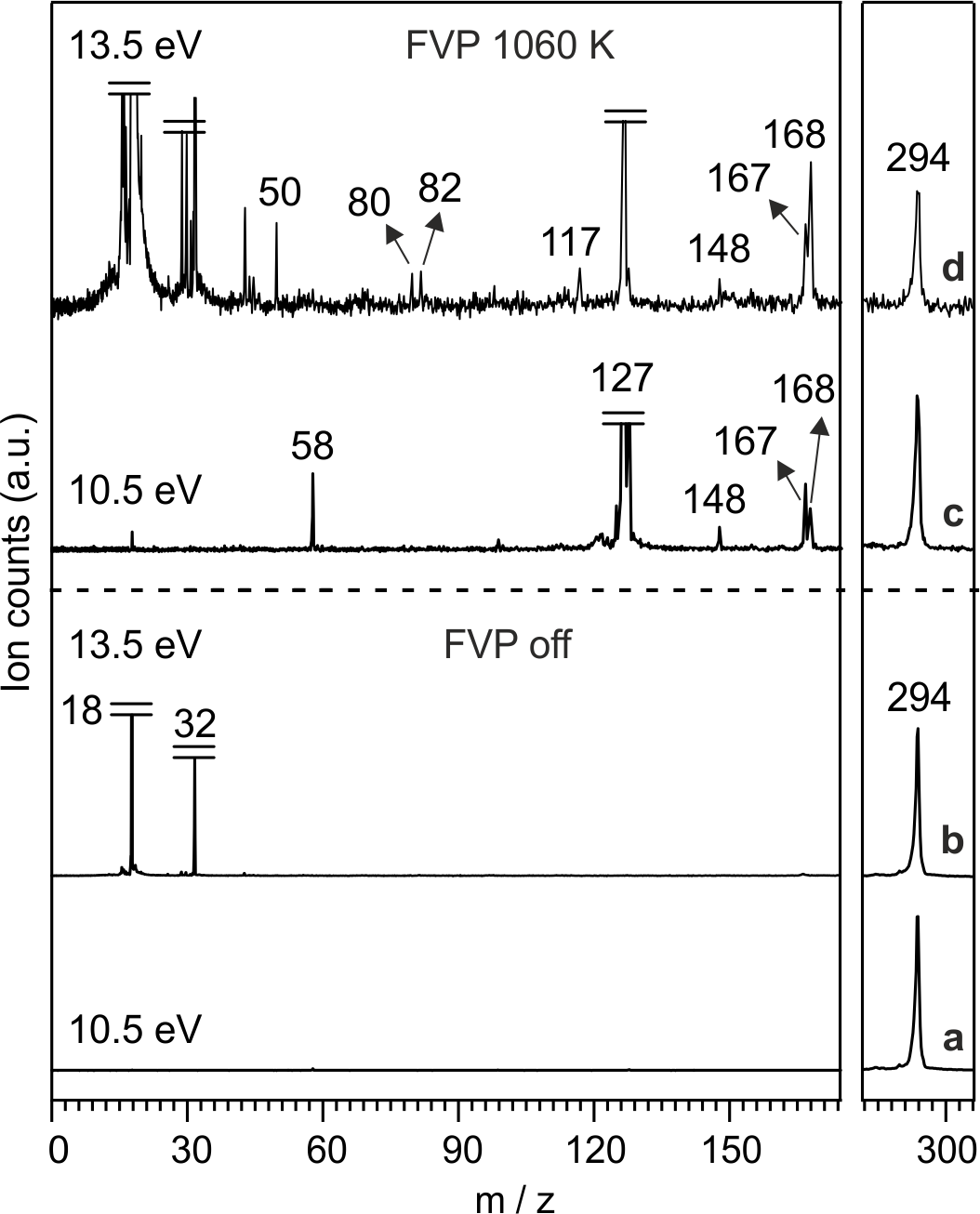


**Figure S6.** Mass spectra of pentafluorophenyl iodide **2b** at RT (pyrolysis off) with hν = 10.5 eV and 13.5 eV (a-b), and upon FVP at 1060 K with hν = 10.5 eV and 13.5 eV (c-d). Peaks corresponding to H_2_O (m/z 18) and O_2_ (m/z 32) as well as detached I atoms (m/z 127) are cut off for clarity. Spectra c-d (full range) are scaled up by a factor of 50 for clarity.


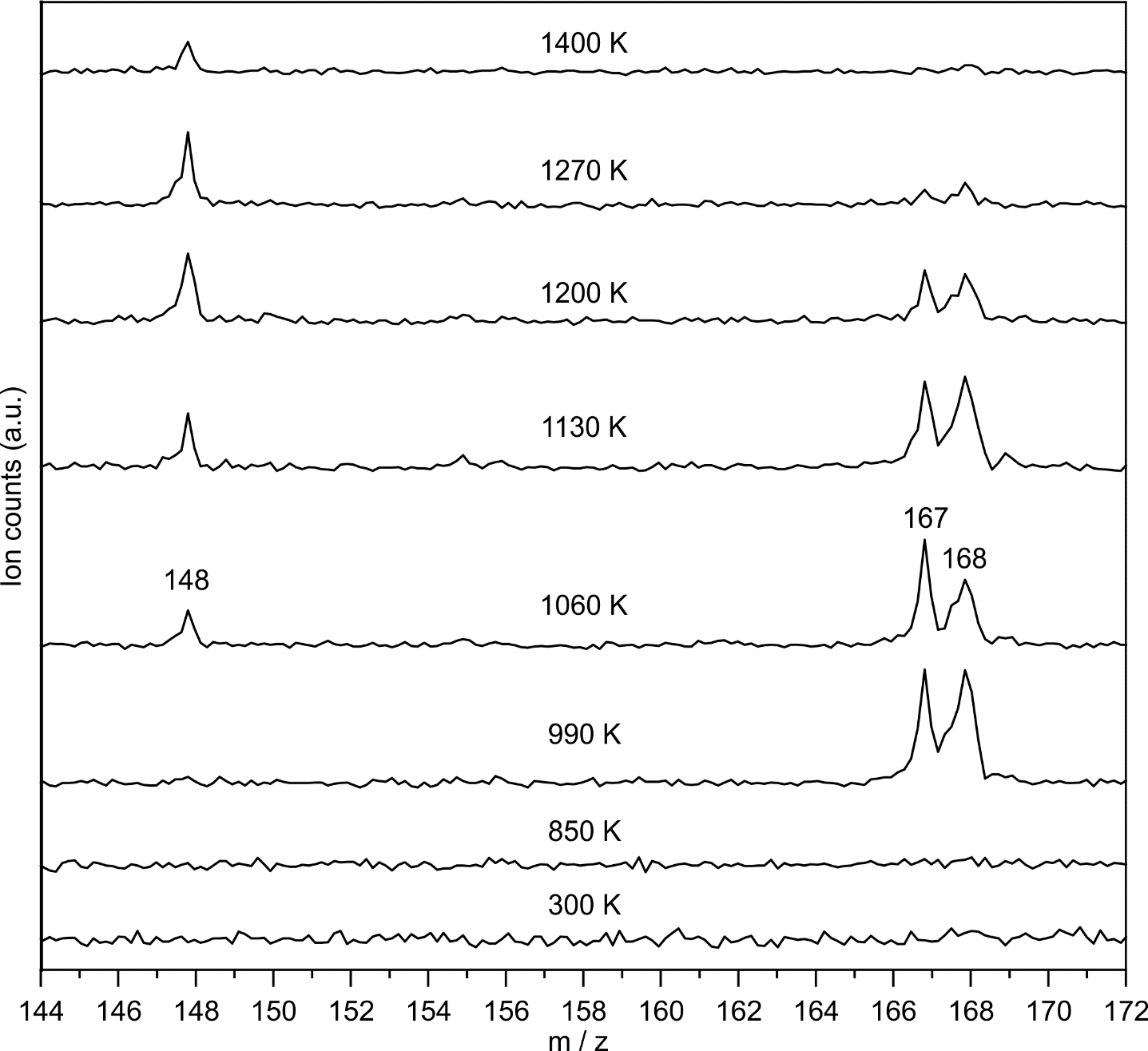


**Figure S7.** Mass spectra recorded upon FVP of pentafluorophenyl iodide **2b** at different temperatures using a fixed photon energy of 10.5 eV. These mass spectra correspond to the whole ion image. The pentafluorophenyl radical **1b** (m/z 167) is formed at 1000–1200 K, but subsequently undergoes F-loss to give tetrafluoro-*o*-benzyne **10b** in the hot reactor. At 1400 K, the yield of **10b** decreases due to its subsequent fragmentation into smaller molecules (*vide infra*).


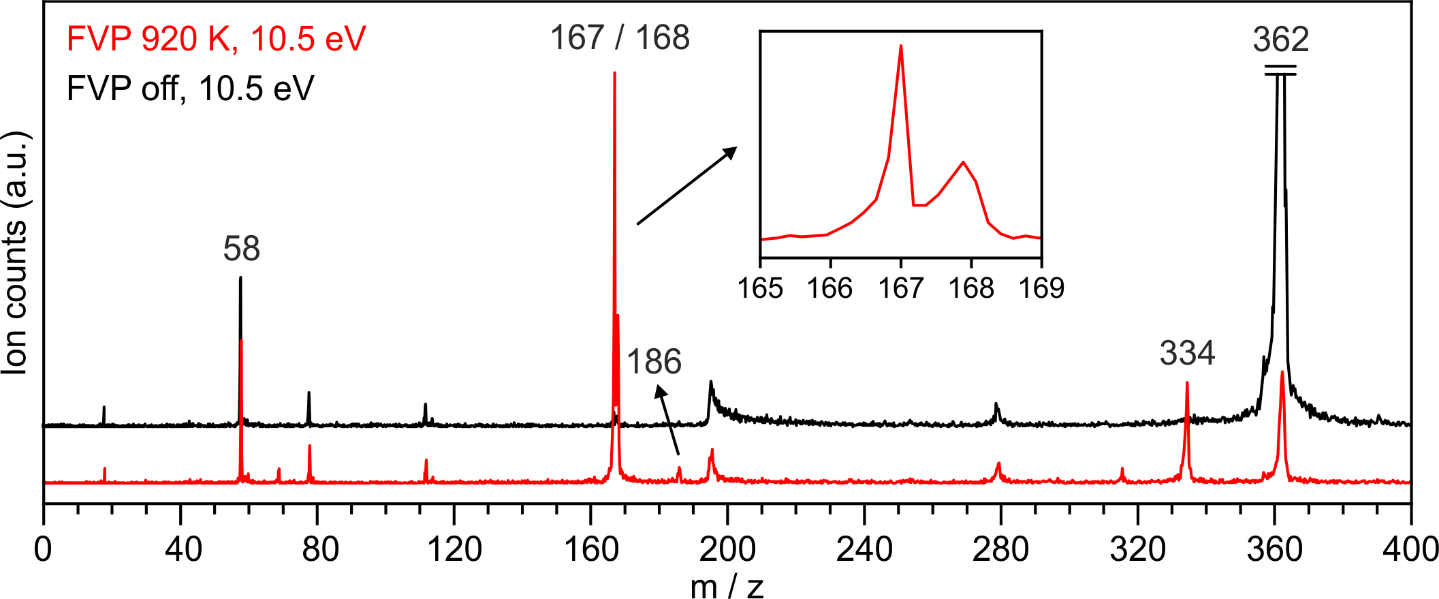


**Figure S8.** Mass spectra of decafluoroazobenzene **3b** at RT (pyrolysis off) and upon FVP at 920 K with hν = 10.5 eV. The peak corresponding to **3b** (m/z 362) is cut off for clarity. The peak at (m/z 334) is assigned to decafluorobiphenyl (m/z 334), formed upon dimerization of pentafluorophenyl radical **1b**, while hexafluorobenzene (m/z 186), obtained upon F-abstraction from radical **1b**, is also detected in traces. Perfluorinated 1,2,3-cyclohexatriene (C₆F₆) is calculated to lie approximately 80 kcal mol⁻¹ above hexafluorobenzene at the B3LYP/6-311+G* level of theory. Therefore, its presence can be ruled out under our experimental conditions. Other peaks arise from contaminants in the sample line, including acetone (m/z 58). Inset shows the expansion of the peaks with m/z 167 and 168, assigned to radical **1b** and pentafluorophenyl benzene **9b**, respectively.

**Fragments observed upon FVP of 2b, tentative assignment and ion counts in brackets. All molecules in both the molecular beam (MB) and background (BG) contribute to the spectra, unless otherwise indicated.**

**300 K (pyro off) and 13.5 eV**: 294 (C_6_F_5_I, 20000), 167 (C_6_F_5_, 200), 82 (?, 70), 80 (?, 50), 45 (C_2_H_5_O, 100), 44 (?, 70), 43 (C_2_F, 350), 32 (O_2_, 15000), 31 (CF, 150), 30 (C_2_H_6_, 350), 29 (?, 450), 26 (C_2_H_2_, 150), 20 (HF, 100), 19 (F, 600), 18 (H_2_O, 120000).

**1060 K and 12.0 eV**: 168 (C_6_F_5_H, 350), 167 (C_6_F_5_, 200), 148 (C_6_F_4_, 100), 128 (HI, 150), 127 (I, 2000), 117 (C_5_F_3_, 100), 82 (?, 100), 80 (?, 100), 69 (CF_3_, 50), 58 (acetone, 50), 50 (CF_2_, 250), 45 (C_2_H_5_O, 100), 43 (C_2_F, 250), 32 (O_2_, 9000), 31 (CF, 200), 30 (C_2_H_6_, 400), 29 (C_2_H_5_, 450), 20 (HF, 200), 19 (F, 650), 18 (H_2_O, 108000).

**1060 K and 13.5 eV**: 294 (C_6_F_5_I, 300), 254 (I_2_, 150), 186 (C_6_F_6_, 45), 168 (C_6_F_5_H, 350), 167 (C_6_F_5_, 210), 148 (C_6_F_4_, 75), 128 (HI, 100), 127 (I, 1800), 117 (C_5_F_3_, 100), 82 (?, 100), 80 (?, 100), 77 (C_6_H_5_, 50), 69 (CF_3_, 50), 58 (acetone, 50), 50 (CF_2_, 250), 45 (C_2_H_5_O, 100), 44 (?, 50), 43 (C_2_F, 250), 32 (O_2_, 9000), 31 (CF, 180), 30 (C_2_H_6_, 400), 29 (C_2_H_5_, 400), 20 (HF, 200), 19 (F, 250), 18 (H_2_O, 108000).

**1060 K and 13.5 eV in the MB**: 294 (C_6_F_5_I, 15), 186 (C_6_F_6_, 15), 168 (C_6_F_5_H, 20), 167 (C_6_F_5_, 130), 148 (C_6_F_4_, 100), 127 (I, 950), 124 (C_4_F_4_, 20), 117 (C_5_F_3_, 70), 93 (C_3_F_3_, 10), 50 (CF_2_, 50), 32 (O_2_, 320), 19 (F, 20), 18 (H_2_O, 1300).

**1400 K and 10.5 eV:** 294 (C_6_F_5_I, 60), 254 (I_2_, 250), 186 (C_6_F_6_, 15), 168 (C_6_F_5_H, 10), 148 (C_6_F_4_, 50), 143 (C_4_F_5_, 15), 128 (HI, 100), 127 (I, 4900), 98 (C_5_F_2_, 25), 86 (C_4_F_2_, 40), 58 (acetone, 190), 18 (H_2_O, 30).

**1400 K and 13.5 eV**: 294 (C_6_F_5_I, 60), 254 (I_2_, 80), 186 (C_6_F_6_, 20), 167/168 (60), 148 (C_6_F_4_, 80), 127 (I, 950), 124 (C_4_F_4_, 20), 117 (C_5_F_3_, 40), 98 (C_5_F_2_, 40), 82 (?, 100), 80 (?, 50), 71 (?, 50), 67 (C_4_F, 200), 57 (?, 100), 50 (CF_2_, 160), 46 (C_2_H_5_OH,100), 45 (C_2_H_5_O, 130), 44 (?, 1000), 43 (C_2_F, 300), 36 (70), 32 (O_2_, 10000), 31 (CF, 200), 30 (C_2_H_6,_ 400), 29 (C_2_H_5,_ 400), 28 (?, 60), 26 (?, 80), 20 (HF, 160), 19 (F, 20), 18 (H_2_O, 111000), 16 (CH_4_, 1000).

**1400 K and 13.5 eV in the MB**: 148 (C_6_F_4_, 50), 127 (I, 800), 67 (C_4_F, 120), 50 (CF_2_, 40), 46 (C_2_H_5_OH, 50), 45 (C_2_H_5_O, 50), 44 (CO_2_, 500), 32 (O_2_, 250), 18 (H_2_O, 1000).

# Velocity Map Imaging

In velocity map imaging, the ions/electrons are dispersed onto a 2D detector according to their momentum. The center of the detector shows a broad distribution, which results from ionization of the background (BG) gas in the chamber, having similar x and y axes contributions. In a molecular beam experiment, the expansion in vacuum yields a narrow velocity distribution of the ions perpendicular to the molecular beam (MB) axis, which is indicated by a narrow line of ionized precursor **2b** (m/z 294) in the panel A, left image, top part. Molecules impinging the chamber retrieve their initial room temperature velocity distribution and are responsible for the background (BG) part of the ion image. Dissociative photoionization (DPI) processes result in kinetic energy release perpendicular to the MB axis. In addition, some reactive intermediates in the MB diffuse away and may collide with the walls of the chamber releasing their excess of thermal energy^[6]^ and undergo secondary reactions like H-abstraction^[18]^ before diffusing back into the ionization volume. These molecules thus only contribute to the BG signal.

Upon FVP at 1060 K, the ion image of m/z 167 shows a large concentration of ions with a narrow velocity distribution, indicative of direct ionization of radical **1b** and negligible contribution of ions formed upon DPI of unreacted precursor **2b** (panel A, center image). In contrast, the right image in panel A shows ions of m/z 168, with a broad room temperature (background) velocity distribution as compared to the middle panel, proving that **9b** is not formed in the MB but rather via collision of the reactive radical **1b** with the chamber walls and hydrogen abstraction from any adsorbed species with a R–H bond. Direct ionization of radical **1b** and negligible DPI is also shown upon FVP of precursor **3b** (panel B, right image). The ms-TPE spectra in the manuscript were recorded within photon energies in which DPI was not detected.

| **A** | **B** |
| --- | --- |
| 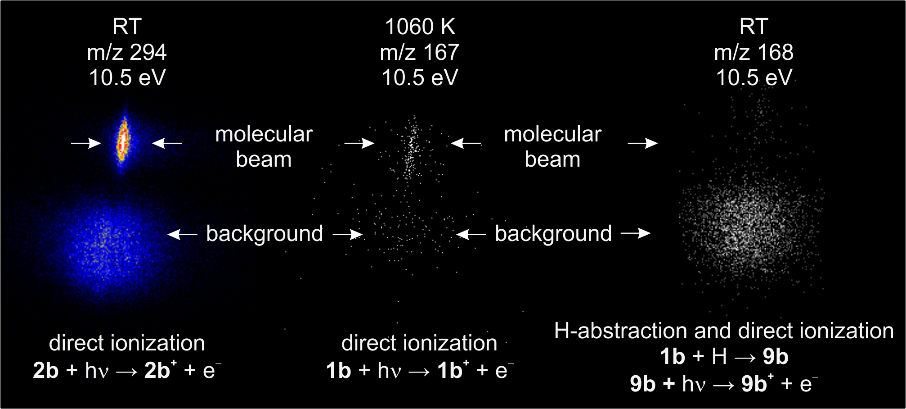 | 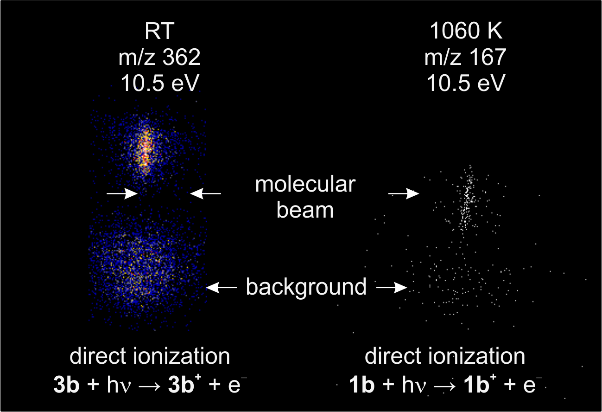 |

**Figure S9.** Panel **A**: selected ion images taken at 10.5 eV photon energy of perfluorophenyl iodide ion **2b^+^** (m/z 294), pentafluorophenyl cation **1b^+^** (m/z 167), pentafluorobenzene **1b^+^** (m/z 167), with pyrolysis on and off. Panel **B**: selected ion images taken at 10.5 eV photon energy of perfluoroazobenzene ion **3b^+^** (m/z 362) and perfluorophenyl cation **1b^+^** (m/z 167) with pyrolysis on and off.

# ms-TPE Spectra of the FVP Products

The MB component contains hot molecules, including radical **1b**, leading to broad ms-TPE spectra with strong hot and sequence band contributions, resulting in a low vibrational resolution.^[6]^ In contrast, the BG is populated with molecules like **9b** rethermalized to RT through wall collisions, which results into better resolved spectra at RT (see Figure S12).


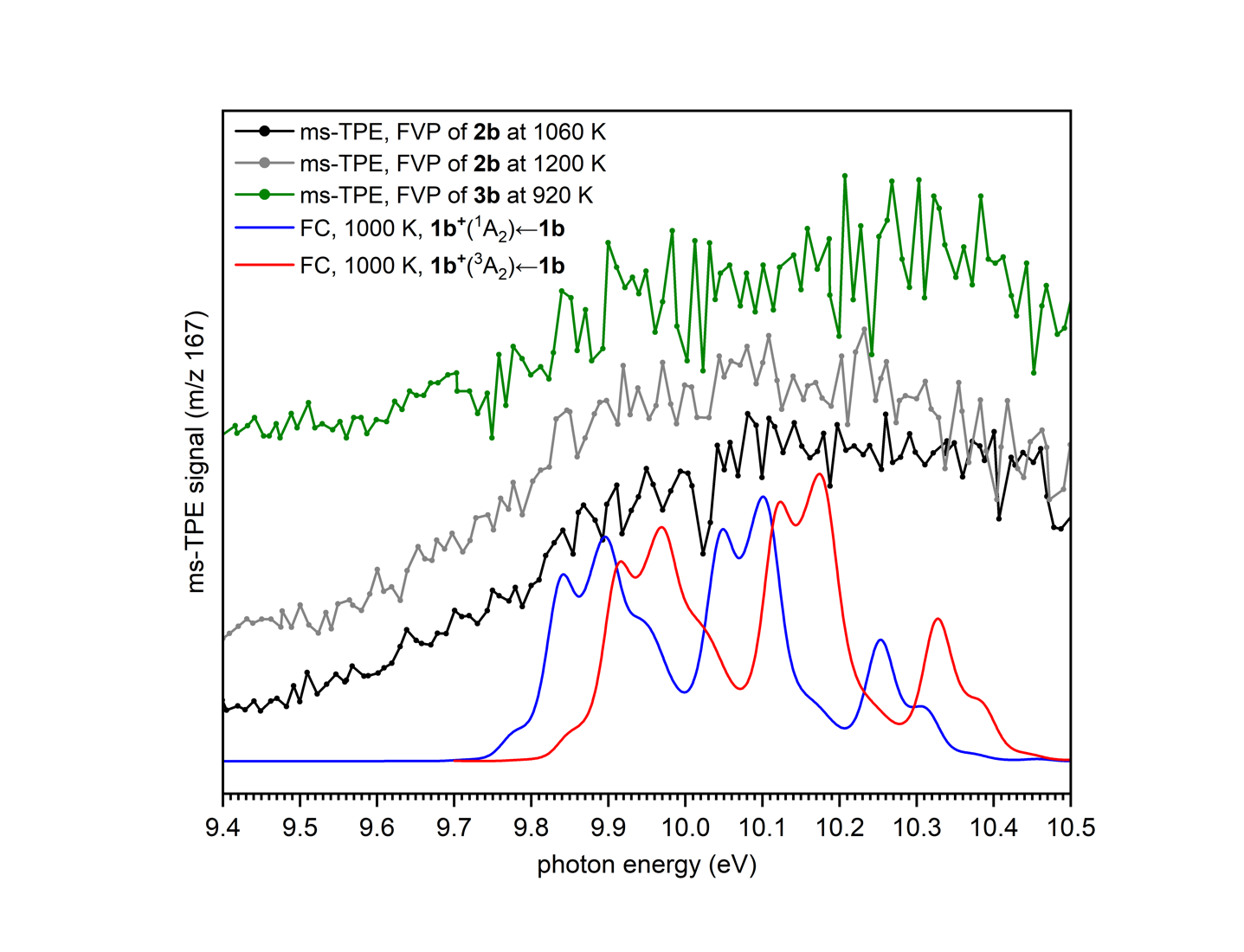


**Figure S10.** ms-TPE spectra of the signal m/z 167, recorded under different experimental conditions: FVP of precursor **2b** at 1060 K (black trace) and 1200 K (grey trace); and FVP of precursor **3b** at 920 K (green trace). The Franck−Condon (FC) simulations at 1000 K of the vibronic transitions of radical **1b** to cation **1b^+^** in its ^1^A_2_ (blue trace) and ^3^A_2_ (red trace) electronic states are shown below. FC simulations are convoluted using 35 meV fwhm Gaussians. The FC transitions at 0 K were calculated using the CASSCF/aug-cc-pVDZ vibrational frequencies and convoluted using 35 meV fwhm Gaussians.


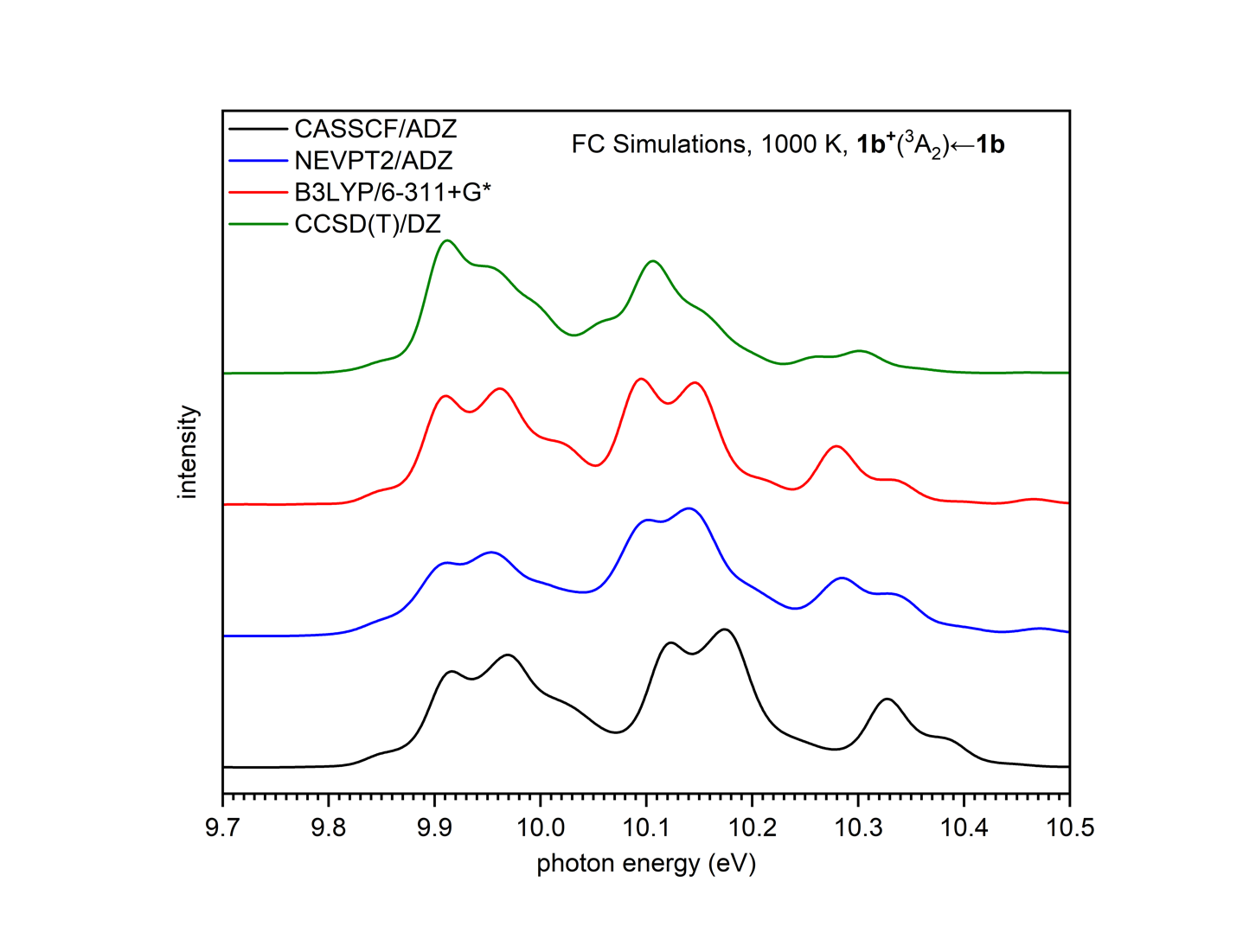


**Figure S11.** Comparison of the Franck−Condon (FC) simulations at 1000 K of the vibronic transition of radical **1b** to cation **1b^+^** in the ^3^A_2_ electronic state using vibrational frequencies calculated with: CASSCF/ CCSD(T)/aug-cc-pVDZ (black trace), NEVPT2/aug-cc-pVDZ (blue trace), B3LYP/6-311+G* (red trace), and CCSD(T)/cc-pVDZ (green trace). FC simulations are convoluted using 35 meV fwhm Gaussians.


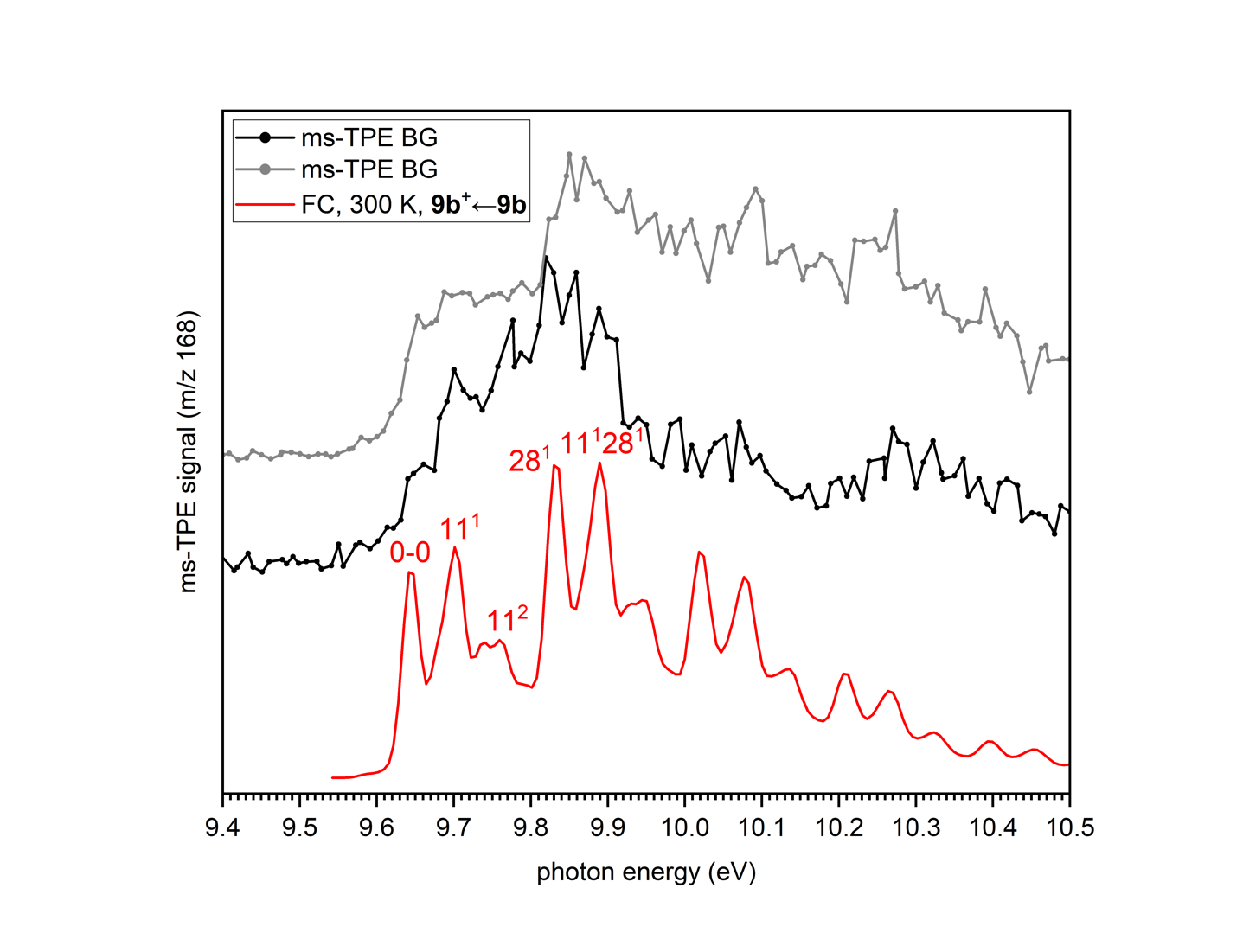


**Figure S12.** ms-TPE spectra of the signal m/z 168, obtained upon FVP of precursor **2b** at 1000 K (black trace) and 1200 K (grey trace), from the BG component of the ion image with an estimated temperature of 300 K. Franck−Condon (FC) simulations at 300 K of the vibronic transitions of **9b** to cation **9b^+^** (red trace) were obtained from the B3LYP/6-311+G* calculated vibrational frequencies and convoluted using 35 meV fwhm Gaussians. The good fit of the experimental and FC simulation spectra justifies our assignment of the formation of **9b** upon hydrogen abstraction.


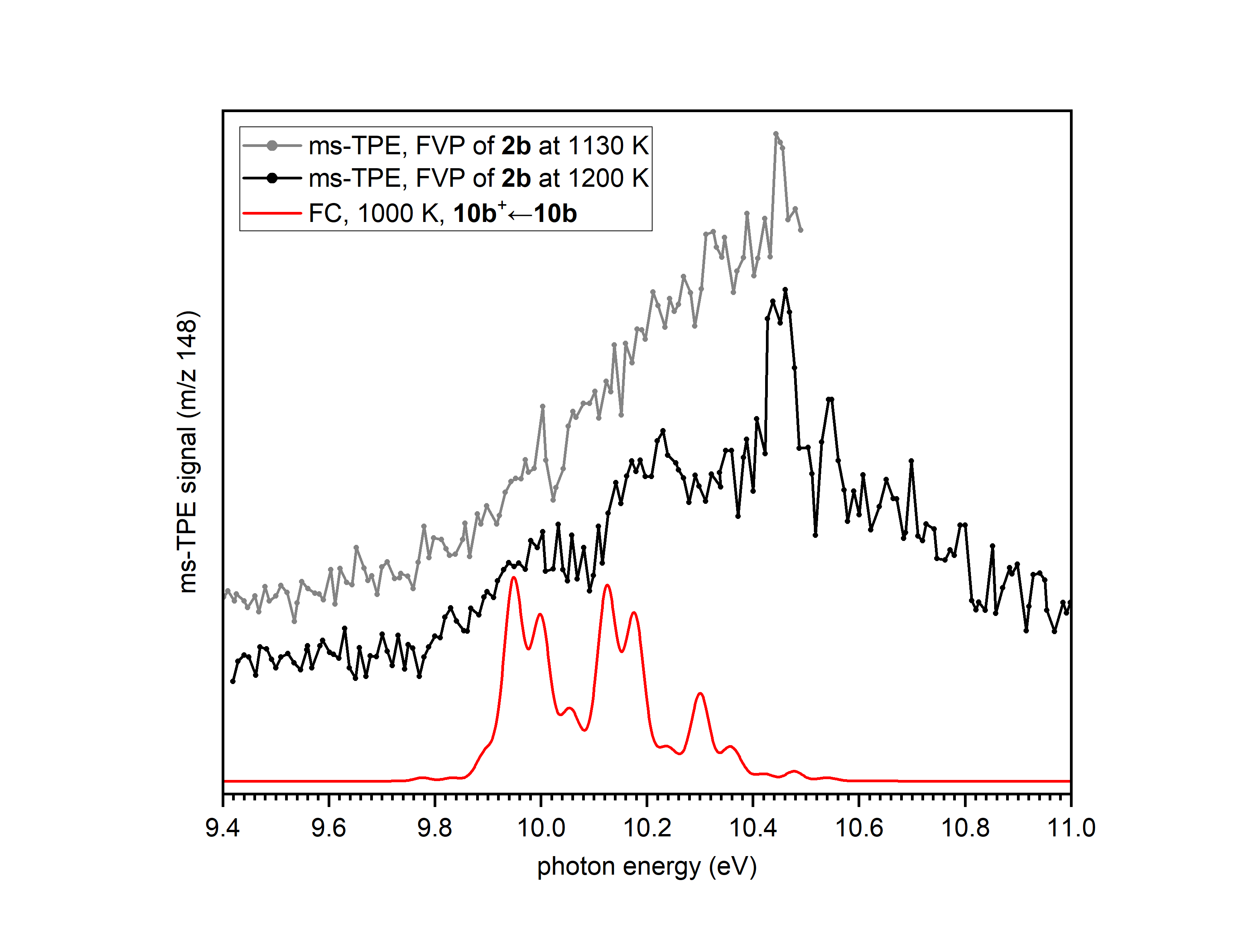


**Figure S13.** ms-TPE spectra of the signal m/z 148, obtained upon FVP of precursor **2b** at 1200 K (black trace, full range) and 1130 K (grey trace, short range). Franck−Condon (FC) simulations at 300 K of the vibronic transitions of tetrafluoro-*o*-benzyne **10b** to its cation **10b^+^** (^2^A_2_) (red trace) were obtained from the B3LYP/6-311+G* calculated vibrational frequencies and convoluted using 35 meV fwhm Gaussians.

Table S3. Calculated Relative Energies and (Adiabatic Ionization Energies) of C_6_F_4_ Isomers.

| **Method^[a]^** |  |  |  |  |  |
| --- | --- | --- | --- | --- | --- |
|  | **10b** | **11b** | **12b** | **13b** | **14b** |
| **ms-TPE (exp.)** | (9.95) | – | – | – | – |
| **B3LYP/6-311+G*** | 0.0 (9.90) | +9.0 (9.31) | – | +36.5 (8.76) | +36.4 (8.70) |
| **CASPT2/ATZ^[b]^** | 0.0 (9.76) | +10.7 (9.35) | **^1^A_g_**: +32.7 (9.68)  **^3^B_1u_**: +33.5 (9.65) | – | – |
| **CCSD(T)/ADZ** | 0.0 (9.88) | +11.9 (9.35) | **^3^B_1u_**: +33.2 (9.72) | +48.2 (8.98) | +48.4 (8.94) |
| **CBS-QB3** | 0.0 (10.05) | +11.3 (9.42) | – | +42.8 (9.11) | +43.3 (9.05) |
| **G4** | 0.0 (9.91) | +11.7 (9.32) | – | +43.5 (9.08) | +43.8 (9.02) |
| **W1BD** | 0.0 (9.99) | +11.6 (9.37) | – | +43.0 (9.13) | +43.5 (9.07) |

[a] Relative energies given in kcal mol^-1^ and adiabatic ionization energies in parenthesis given in eV. [b] Energies computed with CASPT2(7/8,8) over geometries calculated with CASSCF(7/8,8)/aug-cc-pVTZ. *p*-Benzyne **12b** exhibits nearly degenerate singlet (^1^A_g_) and triplet (^3^B_1u_) states.

# Cartesian Coordinates

Geometries were optimized at the CASPT2(6/7,7)/aug-cc-pVTZ level of theory, except for the highly distorted **1b^+^**-^1^A, which was optimized with CCSD(T)/aug-cc-pVDZ. Electronic energies (E) are given in Hartrees.

| **Pentafluorophenyl radical 1b (^2^A_1_)**  E = -726.62071544 | **Pentafluorophenyl cation 1b^+^ (^3^A_2_)**  E = -726.26004816 |
| --- | --- |
| C 0.0000000000 1.2180318072 0.3922562230  C 0.0000000000 1.2249271321 -1.0140806700  C 0.0000000000 0.0000000000 -1.6681279706  C 0.0000000000 -1.2249271321 -1.0140806700  C 0.0000000000 -1.2180318072 0.3922562230  C 0.0000000000 0.0000000000 1.0983548195  F 0.0000000000 -2.3981660260 -1.6818627529  F 0.0000000000 -2.3909771713 1.0807598704  F 0.0000000000 0.0000000000 2.4145210209  F 0.0000000000 2.3909771713 1.0807598704  F 0.0000000000 2.3981660260 -1.6818627529 | C 0.0000000000 1.2091691622 0.4196803100  C 0.0000000000 1.2087956300 -1.0446524821  C 0.0000000000 0.0000000000 -1.7210255242  C 0.0000000000 -1.2087956300 -1.0446524821  C 0.0000000000 -1.2091691622 0.4196803100  C 0.0000000000 0.0000000000 1.1358082885  F 0.0000000000 -2.3777252615 -1.6476061298  F 0.0000000000 -2.3489828130 1.0363992491  F 0.0000000000 0.0000000000 2.4564685522  F 0.0000000000 2.3489828130 1.0363992491  F 0.0000000000 2.3777252615 -1.6476061298 |
| **Pentafluorophenyl cation 1b^+^ (^1^A_2_)**  E = -726.26046437 | **Pentafluorophenyl cation 1b^+^ (^3^B_1_)**  E = -726.25775617 |
| C 0.0000000000 1.2077487299 0.4193218807  C 0.0000000000 1.2094146874 -1.0448220892  C 0.0000000000 0.0000000000 -1.7245848778  C 0.0000000000 -1.2094146874 -1.0448220892  C 0.0000000000 -1.2077487299 0.4193218807  C 0.0000000000 0.0000000000 1.1359997112  F 0.0000000000 -2.3768951429 -1.6469204596  F 0.0000000000 -2.3486430014 1.0376157079  F 0.0000000000 0.0000000000 2.4570882981  F 0.0000000000 2.3486430014 1.0376157079  F 0.0000000000 2.3768951429 -1.6469204596 | C 0.0000000000 1.2738758016 0.3891884100  C 0.0000000000 1.2730239270 -1.0017404101  C 0.0000000000 0.0000000000 -1.6293986842  C 0.0000000000 -1.2730239270 -1.0017404101  C 0.0000000000 -1.2738758016 0.3891884100  C 0.0000000000 0.0000000000 1.0736615466  F 0.0000000000 -2.3857776771 -1.7047061573  F 0.0000000000 -2.3753825641 1.1131079363  F 0.0000000000 0.0000000000 2.3629307908  F 0.0000000000 2.3753825641 1.1131079363  F 0.0000000000 2.3857776771 -1.7047061573 |
| **Pentafluorophenyl cation 1b^+^ (^1^B_1_)**  Eel. = -726.23847021 | **Pentafluorophenyl cation 1b^+^ (^1^A_1_)**  Eel. = -726,20698065 |
| C 0.0000000000 1.2763160474 0.3881522664  C 0.0000000000 1.2678420977 -1.0113981963  C 0.0000000000 0.0000000000 -1.6052666066  C 0.0000000000 -1.2678420977 -1.0113981963  C 0.0000000000 -1.2763160474 0.3881522664  C 0.0000000000 0.0000000000 1.0895993730  F 0.0000000000 -2.3872559707 -1.7105540781  F 0.0000000000 -2.3797418773 1.1039770935  F 0.0000000000 0.0000000000 2.3742062732  F 0.0000000000 2.3797418773 1.1039770935  F 0.0000000000 2.3872559707 -1.7105540781 | C 0.0000000000 1.2301261289 0.3968911190  C 0.0000000000 1.3009196675 -1.0358063730  C 0.0000000000 0.0000000000 -1.4810161946  C 0.0000000000 -1.3009196675 -1.0358063730  C 0.0000000000 -1.2301261289 0.3968911190  C 0.0000000000 0.0000000000 1.0875995436  F 0.0000000000 -2.4156298786 -1.7177881980  F 0.0000000000 -2.3829503173 1.0476312954  F 0.0000000000 0.0000000000 2.4104541751  F 0.0000000000 2.3829503173 1.0476312954  F 0.0000000000 2.4156298786 -1.7177881980 |

| **Pentafluorophenyl cation 1b^+^ (^1^A)**  Eel. = -725.82841955 |
| --- |
| C 0.1733575835 1.0910159486 -0.0411003344  C 1.2736043836 0.2818297687 0.1724614724  C 1.0680059488 -1.1523539480 0.1205065343  C -0.2199629758 -1.6016278847 0.1666185804  C -1.3527440095 -0.9128928017 -0.0818520977  C -1.1192083085 0.5169358932 -0.1593506808  F 0.2821655678 2.3893936578 -0.0912968876  F 2.4798086779 0.7448509362 0.1136822127  F -2.1398228456 1.2833717746 0.0052276002  F -2.5682102992 -1.3191417925 0.0924282798  F 2.0766031480 -1.8919699202 -0.2061106402 |

Geometries were optimized at CASSCF(7/8,8)/aug-cc-pVTZ and energies refined with CASPT2(7/8,8)/aug-cc-pVTZ level of theory. Electronic energies (E) are given in Hartrees.

| **Tetrafluoro-*o*-benzyne 10b (^1^A_1_)**  Eel. = -626.94724843 | **Tetrafluoro-*o*-benzyne Cation 10b^+^ (^2^A_2_)**  Eel. = -626.58870097 |
| --- | --- |
| C 0.0000000000 0.7095033355 0.5815061568  C 0.0000000000 -0.7095033355 0.5815061568  C 0.0000000000 -1.4311809117 -0.5920425789  C 0.0000000000 -0.6245027061 -1.7180108660  C 0.0000000000 0.6245027061 -1.7180108660  C 0.0000000000 1.4311809117 -0.5920425789  F 0.0000000000 2.7368665713 -0.5973047396  F 0.0000000000 1.3199479551 1.7410510278  F 0.0000000000 -1.3199479551 1.7410510278  F 0.0000000000 -2.7368665713 -0.5973047396 | C 0.0000000000 0.6897451452 0.6258315113  C 0.0000000000 -0.6897451452 0.6258315113  C 0.0000000000 -1.4227182890 -0.6017375676  C 0.0000000000 -0.6206698261 -1.7492715768  C 0.0000000000 0.6206698261 -1.7492715768  C 0.0000000000 1.4227182890 -0.6017375676  F 0.0000000000 2.6809055555 -0.5888047229  F 0.0000000000 1.3379649080 1.7315493560  F 0.0000000000 -1.3379649080 1.7315493560  F 0.0000000000 -2.6809055555 -0.5888047229 |
| **Tetrafluoro-*m*-benzyne 11b (^1^A_1_)**  Eel. = -626.93018002 | **Tetrafluoro-*m*-benzyne Cation 11b^+^ (^2^A_2_)**  Eel. = -626.58672266 |
| C 0.0000000000 0.0000000000 1.1208560864  C 0.0000000000 -1.1735256231 0.3817302864  C 0.0000000000 -1.0824764674 -0.9881072519  C 0.0000000000 0.0000000000 -1.7997532310  C 0.0000000000 1.0824764674 -0.9881072519  C 0.0000000000 1.1735256231 0.3817302864  F 0.0000000000 2.3397819204 0.9791591527  F 0.0000000000 0.0000000000 2.4261633595  F 0.0000000000 -2.3397819204 0.9791591527  F 0.0000000000 0.0000000000 -3.0988305892 | C 0.0000000000 0.0000000000 1.2568029882  C 0.0000000000 -1.1545618448 0.4599774247  C 0.0000000000 -0.7301251971 -0.8971126213  C 0.0000000000 0.0000000000 -2.0116875405  C 0.0000000000 0.7301251971 -0.8971126213  C 0.0000000000 1.1545618448 0.4599774247  F 0.0000000000 2.3544468068 0.8736372311  F 0.0000000000 0.0000000000 2.5342456967  F 0.0000000000 -2.3544468068 0.8736372311  F 0.0000000000 0.0000000000 -3.2583652135 |
| **Tetrafluoro-*p*-benzyne 12b (^1^A_g_)**  Eel. = -626.89519576 | **Tetrafluoro-*p*-benzyne 12b (^3^B_1u_)**  Eel. = -626.89390818 |
| C 0.0000000000 1.2421257410 0.6960590997  C 0.0000000000 1.2421257410 -0.6960590997  C 0.0000000000 0.0000000000 -1.2721703154  C 0.0000000000 -1.2421257410 -0.6960590997  C 0.0000000000 -1.2421257410 0.6960590997  C 0.0000000000 0.0000000000 1.2721703154  F 0.0000000000 2.3623889543 1.3720021632  F 0.0000000000 2.3623889543 -1.3720021632  F 0.0000000000 -2.3623889543 -1.3720021632  F 0.0000000000 -2.3623889543 1.3720021632 | C 0.0000000000 1.2145378722 0.6952643889  C 0.0000000000 1.2145378722 -0.6952643889  C 0.0000000000 0.0000000000 -1.3416669676  C 0.0000000000 -1.2145378722 -0.6952643889  C 0.0000000000 -1.2145378722 0.6952643889  C 0.0000000000 0.0000000000 1.3416669676  F 0.0000000000 2.3437660888 1.3600317845  F 0.0000000000 2.3437660888 -1.3600317845  F 0.0000000000 -2.3437660888 -1.3600317845  F 0.0000000000 -2.3437660888 1.3600317845 |
| **Tetrafluoro-*p*-benzyne Cation 12b^+^ (^2^A_u_)**  Eel. = -626.53944511 | **Tetrafluoro-*p*-benzyne Cation 12b^+^ (^4^A_u_)**  Eel. = -626.53913596 |
| C 0.0000000000 1.1996704632 0.7242596388  C 0.0000000000 1.1996704632 -0.7242596388  C 0.0000000000 0.0000000000 -1.3895760348  C 0.0000000000 -1.1996704632 -0.7242596388  C 0.0000000000 -1.1996704632 0.7242596388  C 0.0000000000 0.0000000000 1.3895760348  F 0.0000000000 2.3183184987 1.3237861893  F 0.0000000000 2.3183184987 -1.3237861893  F 0.0000000000 -2.3183184987 -1.3237861893  F 0.0000000000 -2.3183184987 1.3237861893 | C 0.0000000000 1.2009972292 0.7244037214  C 0.0000000000 1.2009972292 -0.7244037214  C 0.0000000000 0.0000000000 -1.3842471654  C 0.0000000000 -1.2009972292 -0.7244037214  C 0.0000000000 -1.2009972292 0.7244037214  C 0.0000000000 0.0000000000 1.3842471654  F 0.0000000000 2.3191531995 1.3243501322  F 0.0000000000 2.3191531995 -1.3243501322  F 0.0000000000 -2.3191531995 -1.3243501322  F 0.0000000000 -2.3191531995 1.3243501322 |

# References

[1] B. Sztaray, K. Voronova, K. G. Torma, K. J. Covert, A. Bodi, P. Hemberger, T. Gerber, D. L. Osborn, *J. Chem. Phys.* **2017**, *147*, 013944.

[2] M. Johnson, A. Bodi, L. Schulz, T. Gerber, *Nucl. Instrum. Methods Phys. Res., Sect. A* **2009**, *610*, 597−603.

[3] A. Antoine John, Q. Lin, *J. Org. Chem.* **2017**, *82*, 9873-9876.

[4] D. W. Kohn, H. Clauberg, P. Chen, *Rev. Sci. Instrum.* **1992**, *63*, 4003-4005.

[5] Q. Guan, K. N. Urness, T. K. Ormond, D. E. David, G. B. Ellison, J. W. Daily, *Int. Rev. Phys. Chem.* **2014**, *33*, 447-487

[6] P. Hemberger, X. K. Wu, Z. Y. Pan, A. Bodi, *J. Phys. Chem. A* **2022**, *126*, 2196-2210.

[7] A. Bodi, B. Sztáray, T. Baer, M. Johnson, T. Gerber, *Rev. Sci. Instrum.* **2007**, *78*, 084102.

[8] B. Sztáray, T. Baer, *Rev. Sci. Instrum.* **2003**, *74*, 3763-3768.

[9] M. J. T. Frisch, G. W.; Schlegel, H. B.; Scuseria, G. E.;, M. A. C. Robb, J. R.; Scalmani, G.; Barone, V.; Petersson, G., H. e. a. A.; Nakatsuji, Revision A.03 ed., Gaussian, Inc., Wallingford CT, **2016**.

[10] R. Ahlrichs, M. Bar, M. Haser, H. Horn, C. Kolmel, *Chem. Phys. Lett.* **1989**, *162*, 165-169.

[11] J. A. Montgomery, M. J. Frisch, J. W. Ochterski, G. A. Petersson, *J. Chem. Phys.* **1999**, *110*, 2822-2827.

[12] D. Kaiser, E. Reusch, P. Hemberger, A. Bodi, E. Welz, B. Engels, I. Fischer, *Phys. Chem. Chem. Phys.* **2018**, *20*, 3988-3996.

[13] B. Chen, D. A. Hrovat, S. H. M. Deng, J. Zhang, X. B. Wang, W. T. Borden, *J. Am. Chem. Soc.* **2014**, *136*, 3589-3596.

[14] A. H. Winter, D. E. Falvey, *J. Am. Chem. Soc.* **2010**, *132*, 215-222.

[15] B. Chen, A. Y. Rogachev, D. A. Hrovat, R. Hoffmann, W. T. Borden, *J. Am. Chem. Soc.* **2013**, *135*, 13954-13964.

[16] H. J. Werner, P. J. Knowles, G. Knizia, F. R. Manby, M. Schutz, *WIREs Comput. Mol. Sci.* **2012**, *2*, 242-253.

[17] V. A. Mozhayskiy, A. I. Krylov, ezSpectrum, http://iopenshell.usc.edu/downloads.

[18] E. Mendez-Vega, W. Sander, P. Hemberger, *J. Phys. Chem. A* **2020**, *124*, 3836-3843.
